# Reduced expression of SET7/9, a histone mono-methyltransferase, is associated with gastric cancer progression

**DOI:** 10.18632/oncotarget.6681

**Published:** 2015-12-19

**Authors:** Yoshimitsu Akiyama, Yuki Koda, Sun-ju Byeon, Shu Shimada, Taketo Nishikawaji, Ayuna Sakamoto, Yingxuan Chen, Kazuyuki Kojima, Tatsuyuki Kawano, Yoshinobu Eishi, Dajun Deng, Woo Ho Kim, Wei-Guo Zhu, Yasuhito Yuasa, Shinji Tanaka

**Affiliations:** ^1^ Department of Molecular Oncology, Graduate School of Medical and Dental Sciences, Tokyo Medical and Dental University, Yushima, Bunkyo-ku, Tokyo 113-8519, Japan; ^2^ Department of Pathology, Seoul National University College of Medicine, Jongno-gu, Seoul 110-799, Korea; ^3^ Division of Gastroenterology and Hepatology, Ren Ji Hospital, School of Medicine, Shanghai Jiao Tong University, Shanghai 200001, China; ^4^ Department of Surgical Oncology, Graduate School of Medical and Dental Sciences, Tokyo Medical and Dental University, Yushima, Bunkyo-ku, Tokyo 113-8519, Japan; ^5^ Department of Surgery, Graduate School of Medical and Dental Sciences, Tokyo Medical and Dental University, Yushima, Bunkyo-ku, Tokyo 113-8519, Japan; ^6^ Department of Human Pathology, Graduate School of Medical and Dental Sciences, Tokyo Medical and Dental University, Yushima, Bunkyo-ku, Tokyo 113-8519, Japan; ^7^ Division of Cancer Etiology, Peking University Cancer Hospital and Institute, Beijing 100142, China; ^8^ Department of Biochemistry and Molecular Biology, Peking University Health Science Center, Beijing 100191, China

**Keywords:** gastric cancer, histone methyltransferase, H3K4me1, SET7/9, SREK1IP1

## Abstract

SET7/9, a histone methyltransferase, has two distinct functions for lysine methylation. SET7/9 methylates non-histone proteins, such as p53, and participates in their posttranslational modifications. Although SET7/9 transcriptionally activate the genes via H3K4 mono-methylation, its target genes are poorly understood. To clarify whether or not SET7/9 is related to carcinogenesis, we studied alterations of SET7/9 in gastric cancers (GCs). Among the 376 primary GCs, 129 cases (34.3%) showed loss or weak expression of SET7/9 protein compared to matched non-cancerous tissues by immunohistochemistry. Reduced SET7/9 expression was significantly correlated with clinical aggressiveness and worse prognosis. Knockdown of *SET7/9* in GC cells markedly increased cell proliferation, migration and invasion. Expression of S*REK1IP1, PGC* and *CCDC28B* were inhibited in GC cells with *SET7/9* knockdown, while matrix metalloproteinase genes (*MMP1, MMP7* and *MMP9*) were activated. SET7/9 bound and mono-methylated H3K4 at the region of the approximately 4-6 kb upstream from the *SREK1IP1* transcriptional start site and the promoters of *PGC* and *CDC28B*. Cell proliferation, migration and invasion, and expression of three *MMPs* were increased in GC cells with *SREK1IP* knockdown, which were similar to those of *SET7/9* knockdown. These data suggest that *SET7/9* has tumor suppressor functions, and loss of SET7/9 may contribute to gastric cancer progression.

## INTRODUCTION

Epigenetic alterations including DNA methylation and covalent histone modifications are known as important mechanisms in carcinogenesis. Aberrant DNA methylation at the CpG island (CGI) promoter regions of tumor suppressor genes (TSGs) has been frequently observed in various cancers [1]. Histone marks of di- and tri-methylation of H3 lysines (K) 4 (H3K4me2 and H3K4me3) of the gene promoter regions are drastically correlated with their gene activation [1, 2]. The promoter and enhancer regions of transcriptionally active genes are marked by mono-methylation of H3K4 (H3K4me1) as well [3]. In contrast, transcriptionally inactive genes are characterized by di- and tri-methylated H3K9 (H3K9me2 and H3K9me3) and tri-methylated H3K27 (H3K27me3) [1, 2].

The histone lysine methyltransferases (KMTs) family has a highly conserved SET domain that is characterized in the Drosophila proteins Su(var) 3-9, Enhancer-of-zeste and Trithorax [2, 4]. SET7/9 (also known as SET7, SET9 and SETD7) containing the SET domain has two distinct functions for lysine methylation. First, SET7/9 has a mono-methyltransferase activity for H3K4, which is associated with transcriptional activation of genes [5, 6]. It has been reported that SET7/9 can activate expression of myogenic differentiation genes through H3K4me1 at their promoter/enhancer regions [7], implying that SET7/9 is involved in cellular differentiation. Second, SET7/9 methylates the lysine in non-histone proteins, such as K372 of p53 and K873 of pRB, and subsequently participates in the posttranslational modifications of these proteins [8–10].

Growing evidences obtained on *in vitro* analyses of SET7/9 functions for methylation of non-histone proteins provide the possibility that SET7/9 exerts tumor suppressor activities through p53 stabilization, pRB activation and DNMT1 degradation [8–11]. We also reported that SET7/9 suppresses SUV39H1 methyltransferase activity by methylating codons 105 and 123 in SUV39H1 in response to DNA damage, subsequently induced genomic instability and inhibited cell proliferation [12]. In contrast, activation of estrogen receptor α (ERα) by SET7/9 may be implicated in the development of hormone-dependent breast cancer [13]. Thus, the functions of SET7/9 are controversial in cancers. There have been no reports on SET7/9 alterations in primary cancers, and hence it remains unknown how SET7/9 contribute to carcinogenesis.

Gastric cancer (GC) is the second leading cause of cancer death in the world [14]. GCs are histologically classified into two major types, intestinal and diffuse, and distinct genetic and epigenetic alterations of tumor-related genes have been shown in both types of GCs [15]. Although somatic mutations and expression changes of the histone modifier genes including HMT ones are known to play critical roles in the pathogeneses of various cancers [2, 4], the relationship between alterations of the histone modifier genes and GCs is unclear.

Here we observed that SET7/9 expression was frequently reduced in GCs. The aim of this study is to characterize the clinicopathologic features of primary GCs with loss or weak SET7/9 expression, and further study the functional significances of SET7/9 alterations in gastric carcinogenesis. In addition, to elucidate a role of SET7/9 as an H3K4 mono-methyltransferase, we searched SET7/9 downstream target genes and then investigated their transcriptional regulation associated with H3K4me1.

## RESULTS

### SET7/9 expression and its clinicopathological relevance in primary GCs

**Figure 1:**
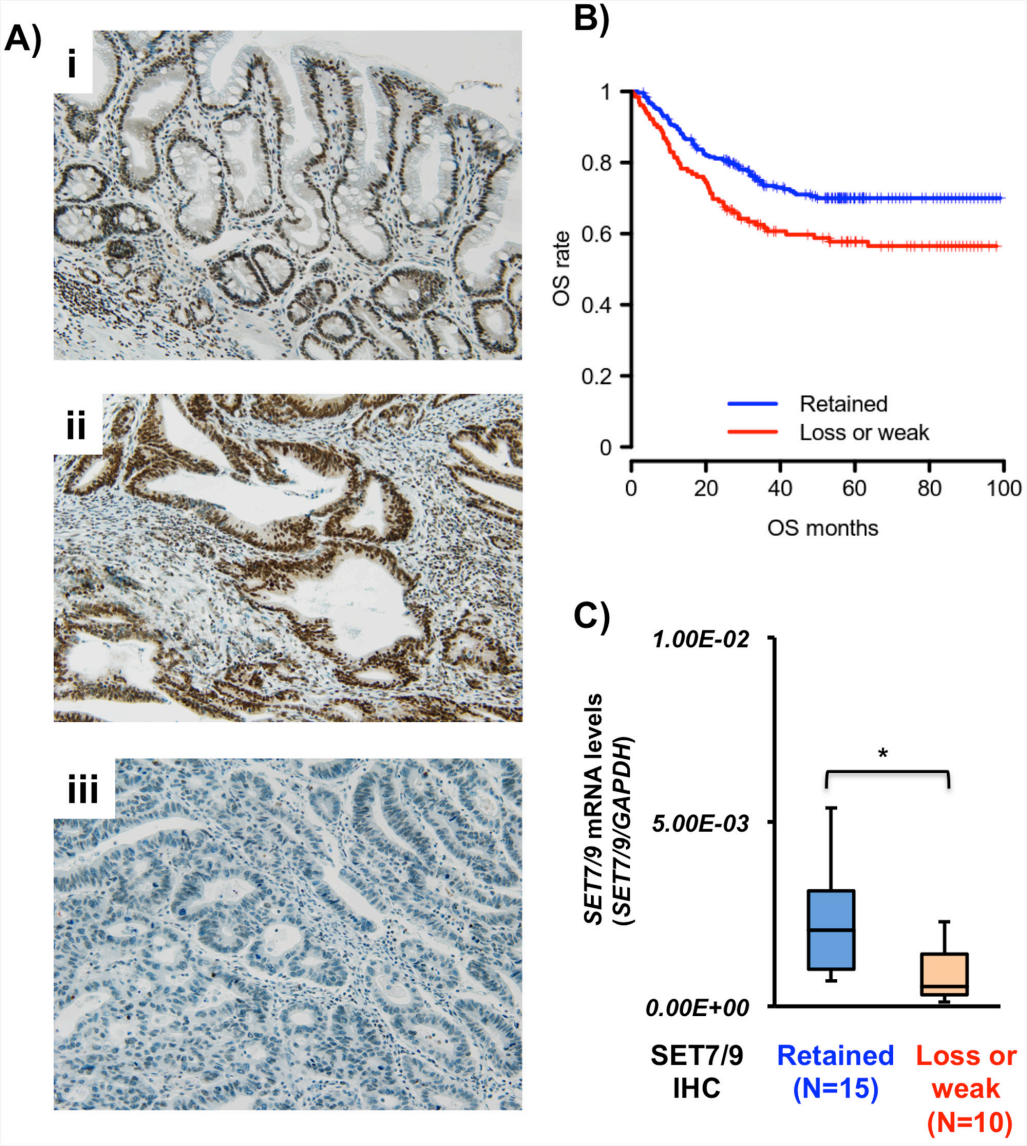
SET7/9 expression in primary GCs **A.** Representative IHC photographs of SET7/9 in primary tissues. Non-cancerous gastric mucosa including intestinal metaplasia exhibited strong SET7/9 expression (i). Positive (ii) and negative (iii) staining of SET7/9 was detected in GCs. Original magnification x100. **B.** Kaplan-Meier curve of overall survival for GC patients with SET7/9 protein expression. The GC patients with loss or weak SET7/9 expression (red, *N* = 129) had a significantly poorer outcome than those with retained SET7/9 (blue, *N* = 247) (*P* = 0.038, logrank test). **C.** qRT-PCR analysis of *SET7/9* expression in 25 primary GC tissues. IHC of SET7/9 was performed in these tissues, which were divided into two groups, SET7/9 protein expression-high (retained, *N* = 15) and -low (loss or weak, *N* = 10). Relative expression was calculated using *GAPDH* expression as an internal control. Mann—Whitney U-test, *P = 0.017.

SET7/9 protein expression was evaluated by immunohistochemistry (IHC), and then graded the SET7/9 expression as weak or loss (expression-low) and retained (expression-high) in primary GCs (Figure 1A). It was noted that SET7/9 protein was strongly expressed in noncancerous gastric epithelial tissues by IHC. Among the 376 GC cases from the formalin-fixed paraffin-embedded (FFPE) tissue microarray, 129 cases (34.3%) showed loss or weak expression of SET7/9 protein compared to matched non-cancerous tissues from the patients.

The relationships between SET7/9 expression and clinicopathological characteristics of 376 FFPE GC cases from the tissue microarray are summarized in Table 1. Loss or weak SET7/9 expression was significantly associated with age (*P* = 0.028), gender (*P* < 0.001), Lauren's classification (*P* < 0.001), Ming's classification (*P* < 0.005), perineural invasion (*P* < 0.001), pT stage (*P* < 0.001), lymph node metastasis (*P* = 0.038), and pTNM stage (*P* < 0.01). The frequency of SET7/9 reduction in advanced GCs was higher than that in early GCs (*P* < 0.054). The patients with GCs showing loss/weak SE7/9 expression exhibited significantly shorter overall survival (OS, *P* = 0.038, Figure 1B) and disease-free survival (DFS, *P* = 0.025, Supplementary Figure S1) than ones with SET7/9-retained GCs with the logrank test. However, SET7/9 expression was not significantly correlated with OS or DFS by multivariate analyses (data not shown).

**Table 1:** Clinicopathological correlations of SET7/9 expression in 376 GCs^1)^

Clinicopathological features	No. of cases	SET7/9 staining^2)^		*P* value^3)^
		Loss or weak (n=129)	Retained (n=247)	
Age				
≤60	198	78 (39.4)	120 (60.6)	0.028*
>60	178	51 (28.7)	127 (71.3)	
Gender				
Female	104	50 (48.1)	54 (51.9)	0.001*
Male	272	79 (29.0)	193 (71.0)	
Lauren's classification				
Intestinal	162	37 (22.8)	125 (77.2)	<0.001*
Non-intestinal (Diffuse/Mixed)	211	92 (43.6)	119 (56.4)	
Ming's classification				
Expanding	41	6 (14.6)	35 (85.4)	0.005*
Infiltrative	335	123 (36.7)	212 (63.3)	
Tumor invasion				
EGC (pT1)	89	23 (25.8)	66 (74.2)	0.054
AGC (pT2-4)	287	106 (36.9)	181 (63.1)	
Lymphatic invasion				
Absent	133	42 (31.6)	91 (68.4)	0.409
Present	243	87 (35.8)	156 (64.2)	
Venous invasion				
Absent	304	103 (33.9)	201 (66.1)	0.72
Present	72	26 (36.1)	46 (63.9)	
Perineural invasion				
Absent	177	46 (26.0)	131 (74.0)	0.001*
Present	199	83 (41.7)	116 (58.3)	
Radicality				
R0	331	108 (32.6)	223 (67.4)	0.063
R1/2	45	21 (46.7)	24 (53.3)	
pT stage				
pT1	89	23 (25.8)	66 (74.2)	0.001*
pT2	58	15 (25.9)	43 (74.1)	
pT3	138	48 (34.8)	90 (65.2)	
pT4	91	43 (47.3)	48 (52.7)	
pT4a	82	39 (47.6)	43 (52.4)	
pT4b	9	4 (44.4)	5 (55.6)	
Lymph node metastasis				
Absent (N0)	143	42 (29.4)	101 (70.6)	0.114
Present	233	87 (37.3)	146 (62.7)	
N0-N1	194	57 (29.4)	137 (70.6)	0.038*
N2-N3	182	72 (39.6)	110 (60.4)	
pTNM stage				
I	111	31 (27.9)	80 (72.1)	0.009*
II	85	24 (28.2)	61 (71.8)	
III	129	51 (39.5)	78 (60.5)	
IV	51	23 (45.1)	28 (54.9)	

1) A total of 376 primary GCs from the tissue microarray were examined by immunohistochemistry.

2) SET7/9 expression levels were divided into two groups, retained and decreased (loss or weak), as shown under Materials and Methods in Supplementary Information.

3) *P* values were determined by Pearson's chi-square test. The Spearman rank correlation analysis was used for pT and pTMN stages. The TMN staging is based on the 7th edition of AJCC (44).

* Statistically significant difference.

We compared the SET7/9 mRNA and protein expression in other 25 primary GC samples (frozen and matched FFPE tissues) by qRT-PCR and IHC analyses, respectively. The expression levels of *SET7/9* mRNA in 10 GCs with low SET7/9 protein expression were significantly lower than those in 15 GCs with retained SET7/9 (*P* = 0.017, Figure 1C).

### Analysis of SET7/9 expression and mutation in cancer cell lines

**Figure 2:**
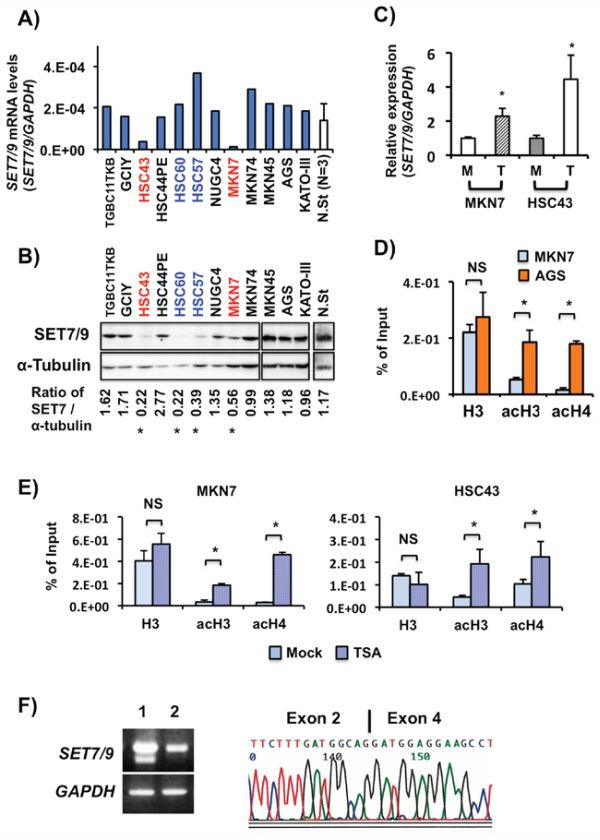
SET7/9 expression in GC cell lines and non-cancerous stomach tissues **A.** *SET7/9* mRNA expression in 12 GC cell lines and three non-cancerous stomach tissues from GC patients. Quantitative RT-PCR was performed using a LightCycler system. The 2^nd^ Derivative Maximum method was utilized for determination of the concentrations, and relative expression was calculated using *GAPDH* expression as an internal control. The averages (blue columns) of two independent experiments for GC cell lines are indicated. The average (white column) and standard deviation (S.D) of the *SET7/9* expression levels in the three non-cancerous stomach tissues (N.St) were also calculated. **B.** WB analysis of SET7/9 protein. a-Tubulin was used as an internal control. The Image-J 1.47v software software (http://imagej.nih.gov/ij/index.html) was used to calculate the SET7/9 protein expression levels. Red, GC cases with weak expression of both SET7/9 mRNA and protein; blue, GC cases with strong *SET7/9* mRNA but weak SET7/9 protein expression. **C.** qRT-PCR analysis of *SET7/9* expression in MKN7 and HSC43 cells after epigenetic drug treatment. MKN7 and HSC43 cells were treated with 0.3 mM trichostatin A (TSA; WAKO, Osaka, Japan) for 24 hrs. M, mock; T, TSA. *P < 0.01. **D.** qChIP assay of the *SET7/9* promoter region in AGS and MKN7 cells exhibiting strong and weak *SET7/9* expression, respectively. ChIP was quantitatively performed with anti-histone H3, and anti-acetylated histone H3 and H4 polyclonal antibodies. Input DNA samples were used as internal controls. **E.** Effects on histone H3 and H4 acetylation in MKN7 cells after TSA treatment. **F.** Longer RT-PCR of *SET7/9* exons 1-8 in TGBC11TKB and KATO-III cells (left). Sequencing analysis of the PCR product of *SET7/9* in TGBC11TKB cells. After subcloning of the RT-PCR product showing an abnormal *SET7/9* transcript in TGBC11TKB cells, sequencing was performed (right). Deletion of exon 3 predicts to encode a truncated SET7/9 protein lacking the SET domain (5,6). We could not detect this predicted small SET7/9 protein (62 aa) in TGBC11TKB cells on WB using anti-SET7/9 antibodies, although wild-type SET7/9 (366 aa) was detected (see Figure 2B).

We examined the SET7/9 expression levels in 12 GC cell lines and three non-cancerous gastric mucosa samples by RT-PCR, quantitative RT-PCR (qRT-PCR) and western blot (WB) (Figures 2A and 2B). Non-cancerous stomach tissues strongly expressed SET7/9 mRNA and protein, which is consistent with data on IHC (Figure 1A (i)). MKN7 and HSC43 showed weak *SET7/9* expression at the mRNA level (Figure 2A). Although expression of SET7/9 protein and mRNA corresponded in most cell lines, HSC60 and HSC57 cells with strong SET7/9 mRNA expression exhibited weak SET7/9 protein. Thus, four of 12 GC cell lines exhibited low SET7/9 protein expression when compared their expression levels of SET7/9 with ones in non-cancerous stomach tissues.

*SET7/9* expression was enhanced in MKN7 and HSC43 cells after treatment with Trichostatin A (TSA) a histone deacetylase inhibitor (HDACi) (*P* < 0.01, Figure 2C). Treatment of SAHA, another HDACi, also showed significantly increased expression of *SET7/9* in MKN7 cells (data not shown). We observed that the histone acetylation levels of H3 and H4 at the *SET7/9* promoter region in AGS cells with strong *SET7/9* expression were much higher than those in its expression-low MKN7 cells by quantitative chromatin immunoprecipitation (qChIP) assay (*P* < 0.01, Figure 2D, and Supplementary Figure S2A). The histone acetylation levels of H3 and H4 were significantly increased in MKN7 and HSC43 cells with TSA treatments (*P* < 0.01, Figure 2E). In contrast, conventional ChIP assays did not show any differences of the H3K4me3, H3K9me3 and H3K27me3 levels between AGS and MKN7 cells (Supplementary Figure S2B). Although SET7/9 expression was increased in MKN7 and HSC43 cells with a de-methylating reagent, 5-aza-dC treatment (*P* < 0.01), CGI hypermethylation at the promoter region of *SET7/9* was not detected in any of the GC cell lines including them and 25 primary GC tissues examined (Supplementary Figures S2C and S2D).

Genetic alterations of *SET7/9* were examined in 12 GC cell lines and 25 primary GC tissues. By long RT-PCR from exons 1-8 and PCR-SSCP, TGBC11TKB cells exhibited a deletion of exon 3 and a C to T transition at the first nucleotide of exon 3 in *SET7/9* mRNA and genomic DNA, respectively (Figure 2F, and Supplementary Figures S2E and S2F), which predicts to encode a truncated SET7/9 protein. Except for TGBC11TKB cells, no mutation was detected in any cases examined.

### The effects of *SET7/9* knockdown in GCs

**Figure 3:**
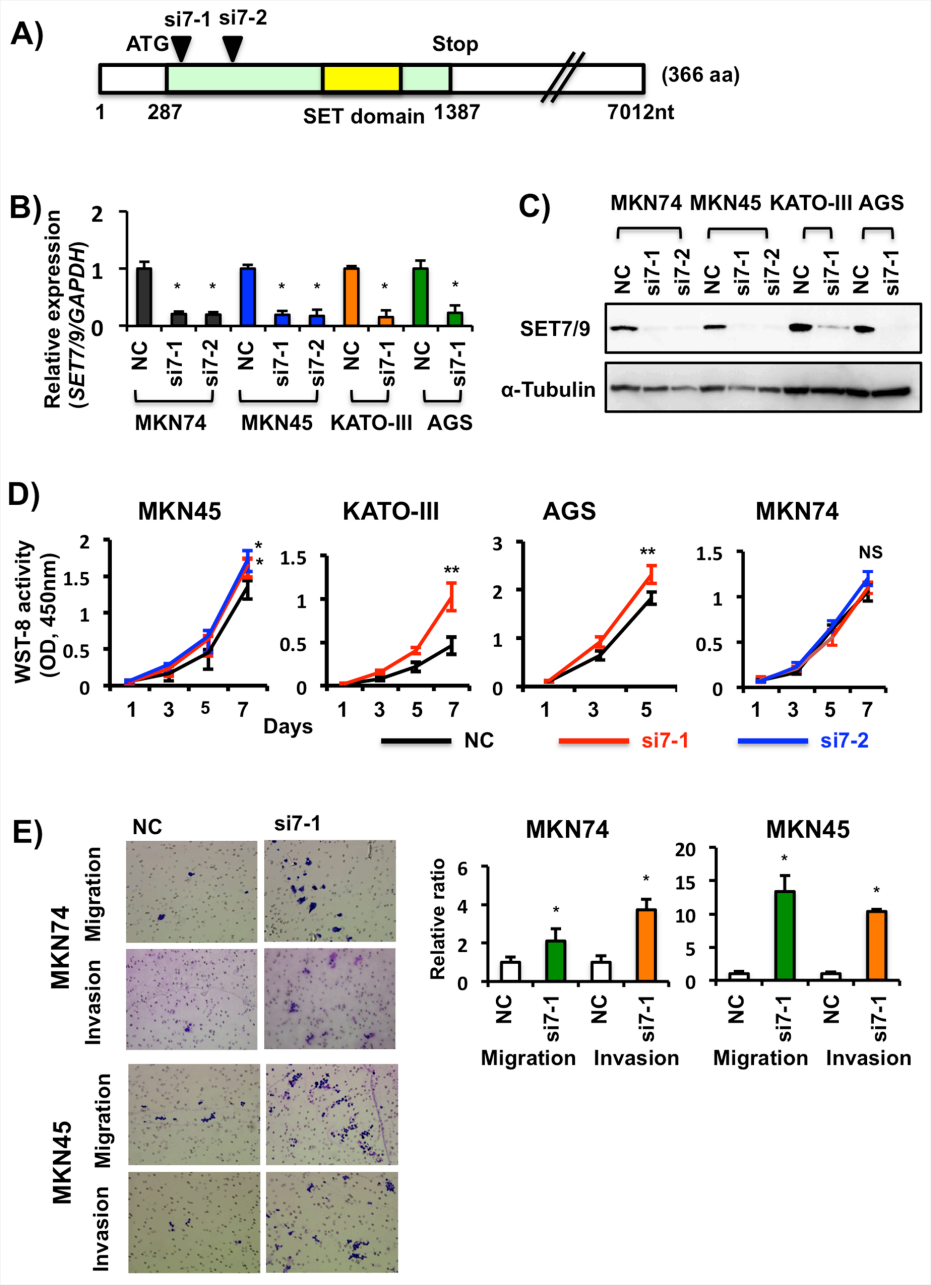
Effects of siRNA-based SET7/9 knockdown in GC cells **A.** Locations of the two *SET7/9* siRNAs used in this study. **B.** qRT-PCR analysis of *SET7/9* expression in four GC cell lines after its siRNA transfection. The average (column) ± S.D (bar) of three independent experiments is indicated. *P < 0.01. **C.** WB analysis of SET7/9 protein expression in GC cell lines. a-Tubulin was used as an internal control. **D.** Cell proliferation assays of four GC cell lines after *SET7/9* knockdown. The number of viable cells was determined using a WST-8 cell proliferation assay kit. Three independent assays were conducted. Representative data are shown and error bars indicate S.D. *P < 0.05; **P < 0.01; NS, not significant. **E.** Cell migration and invasion assays of MKN74 and MKN45 cells after *SET7/9* knockdown. GC cells with *SET7/9* (si7-1) and negative control (NC) siRNA transfection were grown in 24-transwell plates coated with (invasion) or without (migration) matrigel for 18-48 hrs. The total numbers of migrating and invading cells on the membrane were counted, and then relative migration and invasion ratios were calculated. The average (column) ± S.D (bar) of three independent experiments is indicated (*P < 0.01). Photographs show representative fields of migrating and invading cancer cells on the membrane (x100).

Expression of both SET7/9 mRNA and protein were inhibited in four GC cell lines (MKN74, MKN45, AGS and KATO-III) after *SET7/9* siRNAs transfection (Figures 3A-3C). With the exception of MKN74 cells, these three GC cell lines with *SET7/9* knockdown exhibited higher cell proliferation rates compared with ones with negative control siRNA (Figure 3D). Matrigel invasion assays demonstrated that *SET7/9* knockdown significantly enhanced cell invasion (using transwells with matrigels) as well as migration (ones without matrigels) in MKN74, MKN45 (Figure 3E), and AGS cells (Figure 7D, see data on AGS cells with si7-1transfection). Similar effects on cell migration were found in AGS and MKN74 cells with *SET7/9* knockdown on Scratch assay (Supplementary Figure S3).

### Analysis of SET7/9 target genes by microarray

**Figure 4:**
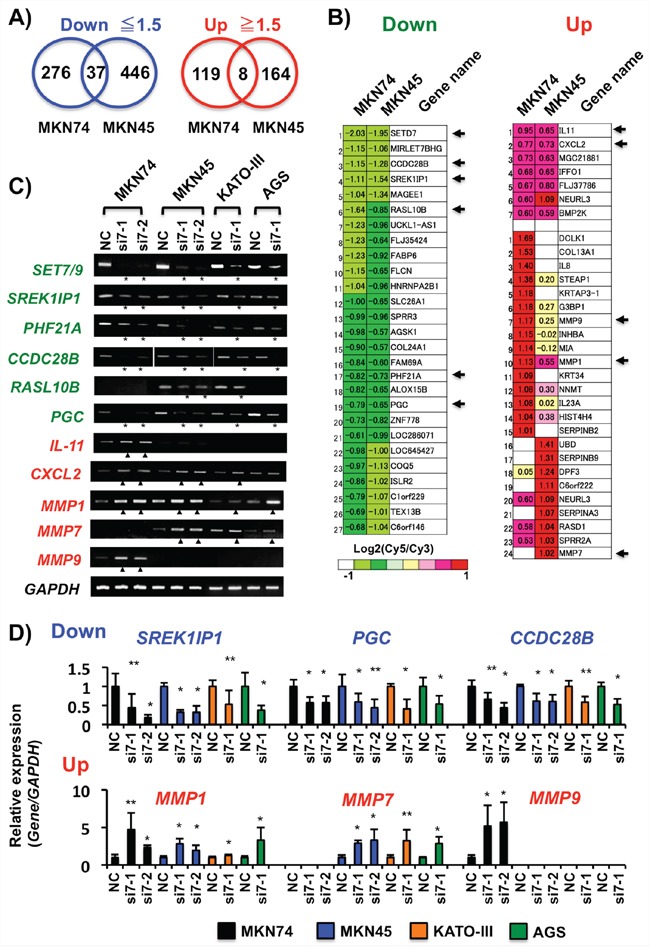
Analyses of the *SET7/9* target genes in GC cells **A.** Venn diagrams of transcriptionally down- and up-regulated gene expression of the genes in MKN74 and MKN45 cells after *SET7/9* knockdown by microarray. The cut-off values of altered expression levels were determined as 1.5-fold change in GC cells with *SET7/9* knockdown compared to ones with negative control transfection. **B.** Heat maps of transcriptionally down- and up-regulated gene expression of the genes in MKN74 and MKN45 cells after *SET7/9* knockdown. The expression levels of the genes are represented by log2 ratios, and cut-off is determined as 1.5-fold changes. Six genes showing reduced expression including *SET7/9* (Green), and five (Red) ones showing increased expression were selected in this study, as shown by arrows. **C.** RT-PCR analyses of the predicted *STE7/9* target genes in 4 GC cell lines after *SET7/9* siRNA transfection. MKN74, MKN45, KATO-III and AGS cells, which exhibited strong *SET7/9* expression, were used in this study. At 48 hrs after transfection, RT-PCR was performed. *GAPDH* was used as an internal control. PCR products showing down- and up-regulation of the *SET7/9* target genes are shown by asterisks and triangles, respectively. **D.** qRT-PCR analyses of the selected genes showing increased and decreased expression by *SET7/9* knockdown in four GC cells. The average (column) ± S.D (bar) of three independent experiments is indicated. *P < 0.01; **P < 0.05.

On microarray analysis, we detected the 313 down- (≤1.5-fold) and 127 up-regulated (≥1.5-fold) probes in MKN74, and 483 and 172 probes in MKN45 cells, respectively, after *SET7/9* knockdown. Moreover, expression of 37 (27 genes) and 8 (7 genes) probes were commonly decreased and increased in these two cell lines, respectively (Figures 4A and 4B). Among them, we selected five decreased (*CCDC28B, SREK1IP1/P18SRP/SFRS12IP1, RASL10B, PHF21A/BHC80* and *PGC*) and two increased genes (*IL-11* and *CXCL2*), by microarray, as differentiation-, splicing-, inflammation-, or tumor-related genes. The expression changes of these seven genes were confirmed in MKN74 and MKN45 cells with *SET7/9* knockdown on conventional RT-PCR and qRT-PCR (Figures 4C and 4D). The expression levels of these genes were also changed in KATO-III and AGS cells after *SET7/9* knockdown. As for the three matrix metalloproteinase genes selected, *MMP1* was activated in all the four cell lines examined, and the expression levels of *MMP9* and *MMP7* were increased in MKN74 and the other three GC cell lines, respectively (Figures 4C and 4D).

**Figure 5:**
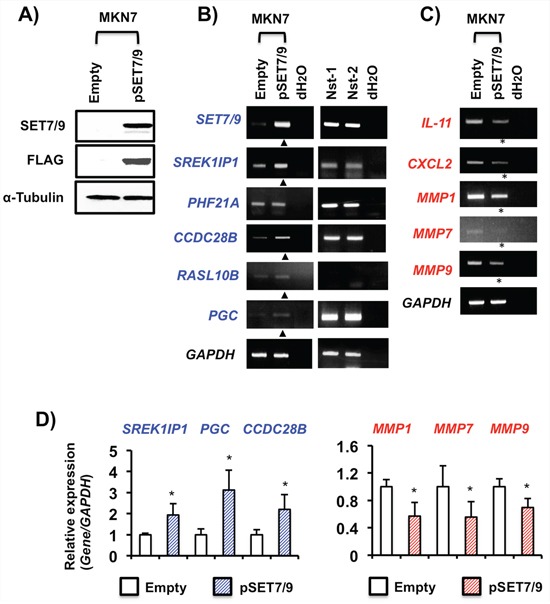
Effects of SET7/9 overexpression in GC cells **A.** WB analysis of SET7/9 protein expression in MKN7 cells. Exogenous SET7/9 protein expression was detected using anti-SET7/9 and anti-FLAG antibodies. Empty: empty vector (p3X-FLAG-CMV^TM^-10), and pSET7/9: wild-type SET7/9. **B.** and **C.** RT-PCR analyses of the *SET7/9* target genes in MKN7 cells after SET7/9 overexpression. The expression levels of the six genes in non-cancerous stomach tissues were also analyzed by RT-PCR. Expressions of the genes showing increased and decreased expression are shown by triangles and asterisks, respectively. **D.** qRT-PCR analyses of *SREK1IP1*, *PGC*, *CCDC28B* and three MMP genes in MKN7 cells exhibiting SET7/9 overexpression. The average (column) ± S.D (bar) of three independent experiments is indicated. *P < 0.01.

On the contrary, *SREK1IP1*, *CCDC28B, RASL10B* and *PGC* were activated in MKN7 cells with exogenous SET7/9 overexpression, while *PHF21A* was not (Figures 5A, 5B and 5D). The *SREK1IP1, PHF21A, CCDC28B* and *PGC* genes were expressed in non-cancerous stomach tissues by RT-PCR (Figure 5B). In contrast, SET7/9 overexpression inhibited expression of *IL-11*, *CXCL2, MMP1, MMP7* and *MMP9* in MKN7 cells (Figure 5C and 5D).

### Identification of the H3K4 mono-methylated regions of SET7/9 target genes

**Figure 6:**
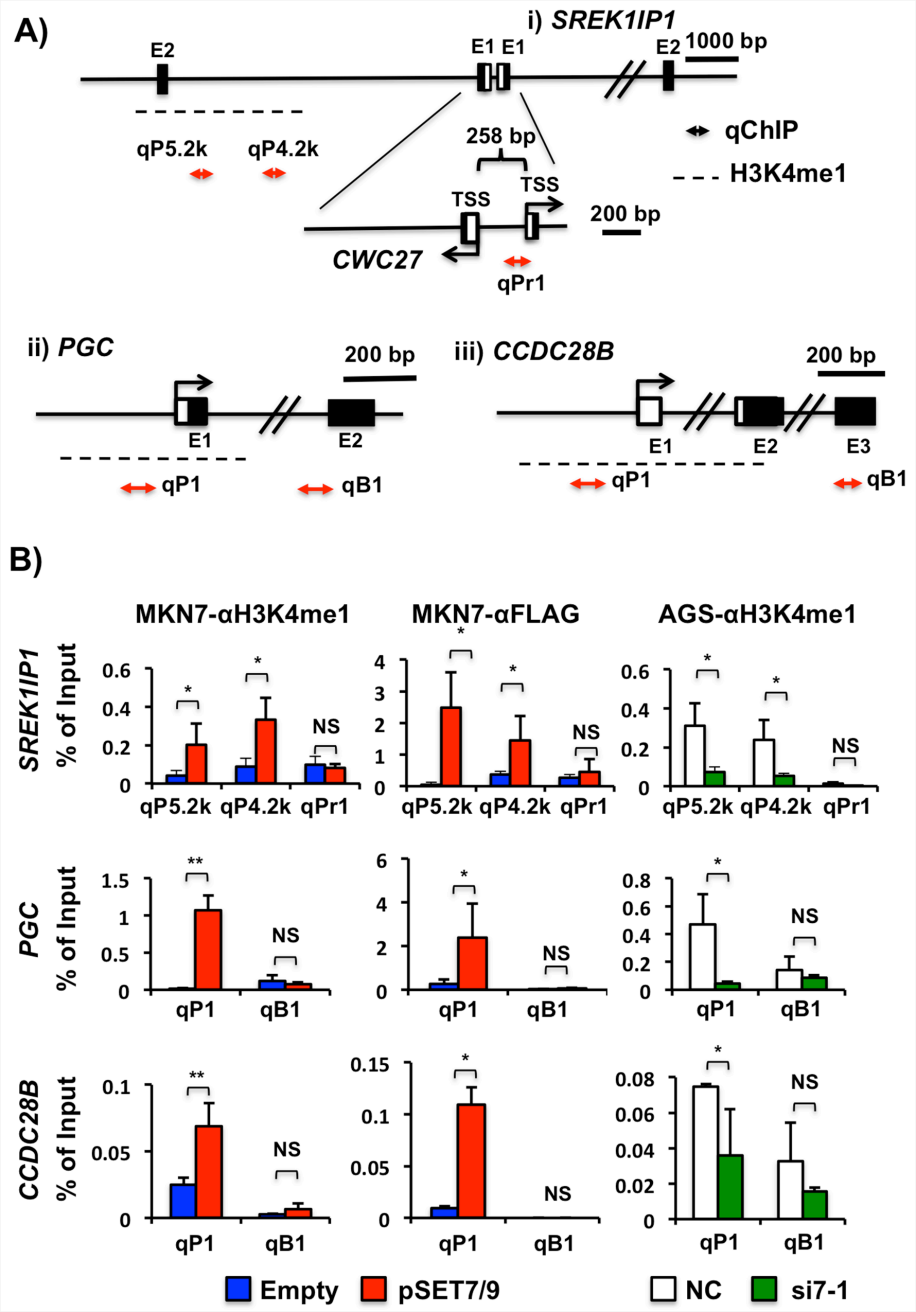
Transcriptional regulatory mechanisms of *SREK1IP1, PGC* and *CCDC28B* via H3K4me1 in GCs **A.** Schematic representation of the 5′-regions of *SREK1IP1*, *PGC* and *CCDC28B*. TSS and closed boxes demonstrate the transcriptional start site and exons, respectively, and horizontal arrows are the qChIP sites in this study. A dotted line indicates an H3K4me1 enrichment region that has been shown by ChIP-seq data on the UCSC database. The *SREK1IP1* and *CWC27* genes are organized in a head-to-head configuration. **B.** The H3K4me1 levels in association with SET7/9 expression. qChIP was performed using anti-H3K4me1 antibodies in MKN7 cells with exogenous SET7/9 overexpression (left) and AGC cells with knockdown of the SET7/9 (right). As SET7/9 was overexpressed in MKN7 cells on transfection of SET7/9-3x FLAG expression vector, ChIP using anti-FLAG antibodies (middle) was performed.*P < 0.01; **P < 0.05; NS, not significant.

We examined whether or not positively activated genes (S*REK1IP1, PHF21A, CCDC28B* and *PGC*) by SET7/9 are correlated with H3K4me1. Because H3K4me1 is known to be marked at transcriptionally active promoter and enhancer regions, we searched the regions by conventional ChIP assays using H3K4me1 antibodies according to the ChIP-seq data on the UCSC Genome Bioinformatics Site (http://genome.ucsc.edu/) (Supplementary Figures S4A, S5A and S6A), and further quantitatively evaluated their histone H3K4me1 levels by qChIP assays (Figure 6A).

Computational analysis showed that the *SREK1IP1* and *CWC27* genes are located in a head-to-head orientation separated by a 258 bp sequence, which predicts a bidirectional promoter in this region. However, MKN7 cells with *SET7/9* overexpression and AGS cells with its knockdown did not show any differences in the H3K4me1 level at this predicted promoter region compared to those with empty/negative control transfection (Figure 6B, qPr1 and Supplementary Figure S4B, Pr1). The ChIP-seq data on the UCSC demonstrates that the region located approximately 4-6 kb upstream from the *SREK1IP1* transcriptional start site (TSS) is enriched with H3K4me1 in GM128 cells, a lymphoid cell line. We observed that overexpression of *SET7/9* in MKN7 cells and knockdown of it in AGS cells increased and decreased the H3K4me1 level at this distal region from *SREK1IP1* TSS, respectively, on conventional ChIP (Supplementary Figure S4B, P4.8k and P5.7k regions) and qChIP (Figure 6B, qP4.2k and qP5.2k regions) assays. Moreover, the H3K4me1 levels at this region tightly corresponded to expression of *SREK1IP1* but not those of *CWC27* (Supplementary Figure S4C).

H3K4me1 at the *PGC* and *CCDC28B* promoter regions was more enriched in MKN7 cells after transfection with the *SET7/9* vector than in ones with the empty vector, while the region at exon 2 and exon 4 in *PGC* and *CCDC28B*, respectively, did not show any difference (Figure 6B and Supplementary Figure S5B). Knockdown of *SET7/9* in AGC cells decreased the H3K4me1 levels at the promoters of these two genes, indicating that the H3K4me1 enrichment at their promoters may be related to their transcriptional regulation. As for *PHF21A*, the H3K4 levels were not changed at the regions within 5 kb upstream from its TSS in GC cells with SET7/9 overexpression and knockdown (Supplementary Figure S6B).

### SET7/9 bound the H3K4 mono-methylated regions in SREK1IP1, PGC and CCDC28B

To further clarify whether or not SET7/9 directly bind the H3K4me1 enrichment region of the genes, we performed ChIP assay using anti-FLAG antibodies in MKN7 with exogenous SET7/9 overexpression. SET7/9 protein specifically bound approximately 4-6 kb upstream from the *SREK1IP1* TSS, and the promoter regions of *PGC* and *CCDC28B* in MKN7 in response to SET7/9 overexpression (see MKN7-αFLAG, Figure 6B, and Supplementary Figures S4B and S5B). Thus, the SET7/9 binding regions in the target genes are consistent with data obtained using anti-H3K4me1 antibodies.

### Functional analysis of the SET7/9 target genes in GC cells

**Figure 7:**
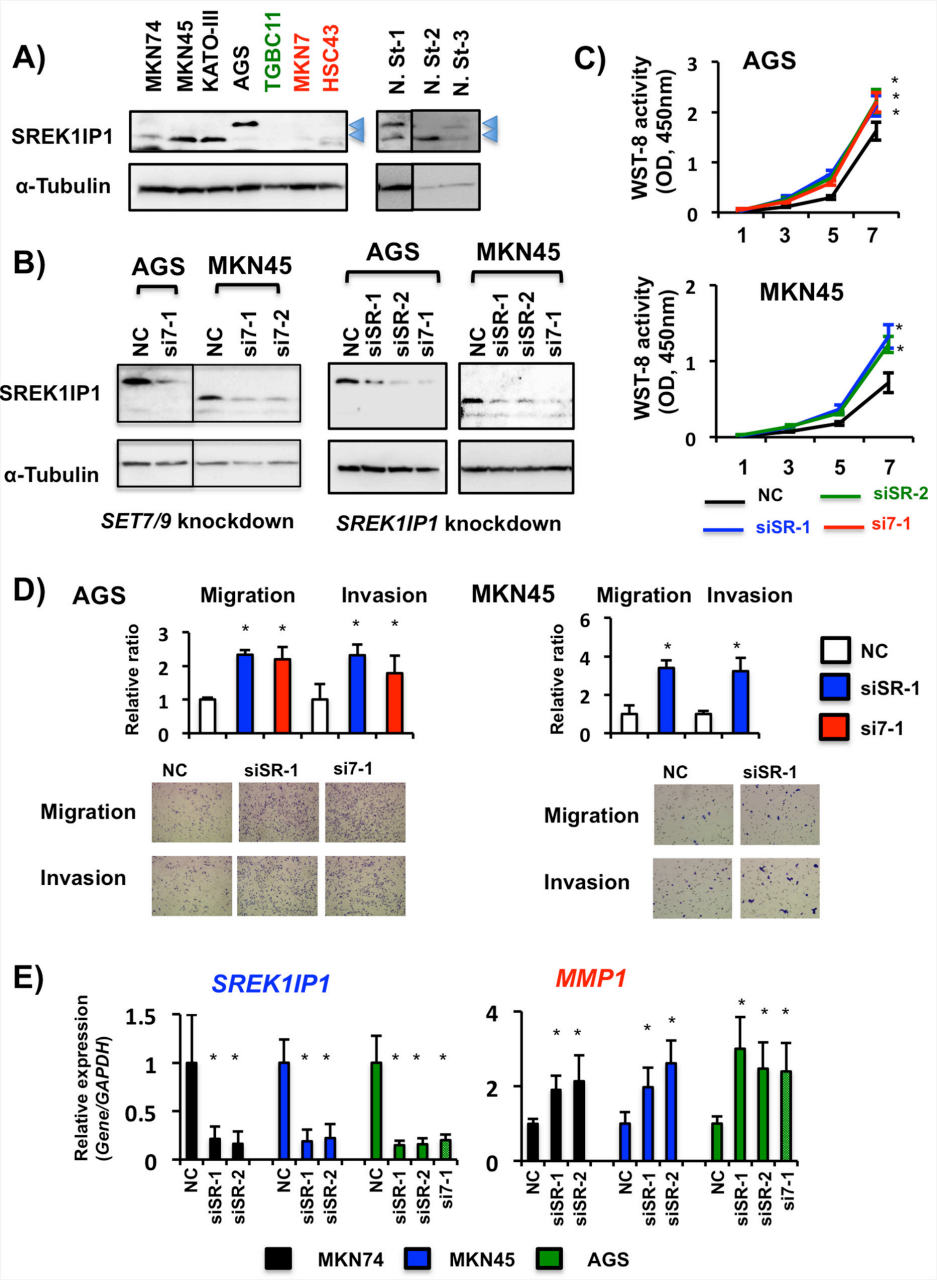
Effects of SREK1IP1 siRNA transfection in GC cells **A.** Endogenous SREK1IP1 protein expression levels in seven GC cell lines and three non-cancerous stomach tissues. Although the size of SREK1IP1 protein is known to be 18kDa, two different sizes (approximately 18kDa and 20kDa, arrowheads) of SREK1IP1 protein were detected on WB. **B.** WB analyses of SREK1IP1 protein expression in AGS and MKN45 cells after *SET7/9* (left, si7-1 and si7-2) and *SREK1IP1* (right, siSR-1 and siSR-2) siRNA transfection. Knockdown of each gene inhibited expression of the predicted (18kDa) and large size (20kDa) proteins in MKN45 and AGS cells, respectively. **C.** Cell proliferation assays after *SREK1IP1* knockdown in AGS and MKN45 cells. To compare the effects on SREK1IP1 and SET7/9, we also performed knockdown of *SET7/9* (si7-1) in this study. Three independent assays of each knockdown were conducted. Representative data are shown and error bars indicate S.D. *P < 0.05. **D.** Matrigel cell invasion and migration assays of AGS and MKN45 cells. The total numbers of migrating and invading cells on the membrane in at least three fields were counted. The average (column) ± S.D (bar) of three independent experiments is indicated (*P < 0.01, top). Photographs show representative fields of migrating and invading cells on the membrane (bottom). **E.** The effects of *SREK1IP1* knockdown by its siRNA transfection and the resultant *MMP1* expression were quantitatively analyzed by qRT-PCR. The average (column) ± S.D (bar) of three independent experiments is indicated (*P < 0.01).

To determine which functions of the genes detected are similar to those of *SET7/9* in GCs, we evaluated the effects on cell migration in AGS cells. We conducted siRNA-based knockdown of *SREK1IP1, PHF21A, CCDC28B* and *PGC* in AGS cells (Supplementary Figures S7A and S7B). At 18hrs after transfection, cell migration was faster in AGS cells with *SREK1IP1* siRNA than in those with a negative control and the other three siRNAs on scratch assay (Supplementary Figure S7C). Knockdown of *SREK1IP1* in AGS cells enhanced cell proliferation rate (see Figure 7C), while knockdown of *PHF21A, CCDC28B* and *PGC* did not (data not shown).

### The biological significance of SET7/9 and SREK1IP1 in GC cell lines

We analyzed the relationship between *SET7/9* and *SREK1IP1* in GC cells. At least two different sizes of SREK1IP1 protein, approximately 18kDa and 20kDa, were detected in GC cell lines and non-cancerous stomach tissues by WB (Figure 7A), both of which expression were decreased by *SET7/9* and *SREK1IP1* knockdown in GC cells (Figure 7B). *SREK1IP1* siRNA transfection in AGS and MKN45 cells promoted cell proliferation, migration and invasion (Figures 7C and 7D). Importantly, the relative effects on cell proliferation, migration and invasion in AGS cells with *SREK1IP1* knockdown were similar to in those with *SET7/9* knockdown.

To clarify the molecular mechanisms underlying cell invasion through *SREK1IP1* and *SET7/9*, we examined expression of *CXCL2, IL-11* and three *MMPs* in MKN45 and AGS cells. After *SREK1IP1* siRNA transfection, *MMP1* expression was enhanced in these GC cells, and expression of *MMP7* and *MMP9* was elevated in AGS and MKN74 cells after *SREK1IP1* knockdown, respectively, which are the same effects as SET7/9 siRNAs in these cells (Figure 7E and Supplementary Figure S8A). Nevertheless, expression of *IL-11* and *CXCL2* were not activated by *SREK1IP1* knockdown. Knockdown of *PHF21A*, *CCDC28B* and *PGC* in MKN74 and AGS cells did not induce any expressional changes of three *MMPs* (Supplementary Figure S8B). Thus, the target genes of *SREK1IP1* in GC cells partially resembled to those of *SET7/9*.

### *SREK1IP1* expression analyses in GCs

We divided the 12 GC cell lines into two groups, i.e., SET7/9 alteration -positive (group A; SET7/9-mutated TGBC11TKB cells and four cell lines with low SET7/9 expression) and -negative (group B; remaining seven cases with high SET7/9 expression), and then compared the *SREK1IP1* expression levels between them by qRT-PCR. The average *SREK1IP1* expression level in the group A was significantly lower than that in the group B (*P* = 0.033, Supplementary Figure S8C). As for the 25 primary GCs, GC cases with loss or weak SET7/9 protein expression showed a tendency of weaker *SREK1IP1* expression compared with ones with retained SET7/9 expression (*P* = 0.073, Supplementary Figure S8D).

## DISCUSSION

In this study, we observed frequent SET7/9 reduction in primary GCs. Our data on SET7/9 and its target gene analyses demonstrated that *SET7/9* knockdown in GC cells significantly promoted to cell proliferation, migration and invasion, and enhanced MMPs expression. We also observed that SET7/9 transcriptionally activated *SREK1IP1, CCDC28B* and *PGC* expression through H3K4me1. These data indicate that SET7/9 has tumor suppressive functions in GCs.

The characteristics of GCs with low SET7/9 expression in our study were perineural invasion-positive, Stage III-IV and lymph node metastasis-positive. Frequent SET7/9 reduction was preferentially found in diffuse and infiltrating type GCs, which are reported to be more malignant than intestinal type GCs [16]. It has been observed that *SET7/9*-knockout mice were viable and fertile, and did not develop tumors [17, 18]. Hence, absence of SET7/9 expression may be late event in gastric carcinogenesis and implicated in the clinical aggressiveness of GCs. In addition, reduced SET7/9 expression was significantly associated with shorter OS and DFS of our GC patients. Among the histone modification genes, overexpression of SUV39H1 and EZH2 has been suggested to correlate with poor prognosis in GCs [19, 20]. Reduced SET7/9 expression could be also used as a biomarker for predicting poor survival in GC patients.

The expression levels of *SET7/9* mRNA significantly corresponded to it its protein expression in this study. *SET7/9* was enhanced in MKN7 and HSC43 cells with treatment of HDACi and de-methylation reagents, indicating that reduced *SET7/9* may be caused by epigenetic changes at the mRNA levels in GC cells. *SET7/9* contains a dense CGI at the promoter region. The characteristics of genes silenced by CGI hypermethylation at the gene promoter region are generally known to be restoration of expression on 5-aza-dC but not TSA treatment [1], and distinct histone methylation patterns of H3K4 and H3K9 [21]. Nevertheless, neither CGI hypermethylation nor histone methylation patterns at the *SET7/9* promoter region were found in GC cells with strong and low SET7/9 expression. Although we could not deny the possibility of CGI hypermethylation except the promoter region, such as a CpG shore and gene body region, the significant relationships between *SET7/9* expression and histone acetylation patterns of histone H3 and H4 at its promoter region were shown in GC cells. Thus, epigenetic changes through decrease of the histone acetylation levels of *SET7/9* should be considered as the factors associated with its gene silencing.

Recently, frameshift mutation of *SET7/9* was reported in a castration-resistant prostate cancer by whole transcriptome sequencing [22]. We also observed an out-of-frame deletion in TGBC11TKB cells. Somatic mutations of the HMT genes including SET7/9 have been shown in several types of cancers according to the COSMIC database [23]. Hence, somatic mutations of *SET7/9* as well as loss of its expression may contribute to the development of diverse cancers.

Our data on knockdown and overexpression of SET7/9 demonstrate that SET7/9 suppressed *CXCL2, IL-11* and three *MMP* genes in GC cells, which were shown to promote not only cancer cell motility and invasion but also metastasis [24–26]. Most importantly, the phenotypes of SREK1IP1 functions were similar to those of SET7/9. First, the endogenous expression patterns of *SREK1IP1* and *SET7/9* were similar in primary GCs as well as GC cell lines. Second, knockdown of *SREK1IP1* in GC cells promoted cell growth, migration and invasion, and increased transcriptional regulation of *MMP1, MMP7* and *MMP9*. Thus, it is possible that SREK1IP act as a TSG as well as SET7/9, and they inversely suppressed three *MMPs* expression in GCs. Overexpression of these three MMPs has been reported in advanced GCs [25, 27]. MMP1 overexpression was also correlated with peritoneal metastasis and lymph node metastasis of GCs [27]. Therefore, a *SET7/9*-*SREK1IP1*-*MMPs* axis may be involved in GC progression.

Reduced SREK1IP1 expression was strongly associated with cell proliferation and invasiveness in our study, suggesting that SREK1IP1 may act as a TSG as well as SET7/9 in GCs. Somatic frameshift mutations at the (A)10 repeat site in *SREK1IP1* exon 3 have been frequently detected in CRCs showing severe microsatellite instability [28]. It is likely that absence of SREK1IP1 is caused by not only loss of SET7/9 but also genetic alterations as a target of MSI. SREK1IP1 is predicted to participate as a splicing regulator [29], because overexpression and knockdown of *SRrp86*, a SREK1IP1 interacting protein, resulted in the alternative splicing of various genes, such as *c-Jun* and *IκBβ*, in cancer cells [30]. Further studies are necessary to clarify whether or not SREK1IP1 serves in the splicing process of genes.

The active histone marks of H3K4me1 and H3K27 acetylation (H3K27ac) are enrichments at the region from 4-6 kb upstream of *SREK1IP1* TSS on the UCSC Genome Bioinformatics Site. In our study, overexpression and knockdown of *SET7/9* in GC cells demonstrated that H3K4me1 enrichment at this distal region corresponded to the *SET7/9* expression level. It has reported that Set7/9 was recruited to enhancers of the genes and mono-methylated H3K4 in a MyoD-dependent gene activation manner during muscle differentiation [31]. Therefore, the enrichment of H3K4me1 levels at this region may be important for transcriptional activation of *SREK1IP1* by SET7/9. The enhancer regions of several genes are shown within the introns of neighboring genes [32]. For example, the enhancer region of the *SHH* gene for limb development is located within intron 5 of *Lmbr1*, where is more than 1 Mb far from the *SHH* promoter region [33]. Indeed, the distal region associated with *SREK1IP1* expression is located within intron 1 of *CWC28B*. Thus, it is possible that the H3K4me1 enrichment region of *SREK1IP1* detected in our ChIP assay may act as a transcriptional enhancer.

As for other two genes activated by SET7/9, *PGC* has widely been considered as a differentiation marker of gastric epithelial cells, and encodes pepsinogen C (PG-II), which is secreted from antral glands and corpus chief cells in the stomach and duodenal glands [34]. Although it remain uncertain the functions of CCDC28B, its was reported that depletion of *CCDC28B* in a ciliated human cell line, hTERT-RPE, established from retinal pigmented epithelial cells, resulted in defective ciliogenesis [35], suggesting that CCDC28B may paly a role in cellular differentiation. Since several pathological groups reported the ciliated metaplasia that is implicated in gastric ciliogenesis [36], it is important to analyze the relationship between *CCDC28B* and ciliated metaplasia further. Our data further support the observations that SET7/9 and H3K4me1 is correlated with cell-type- and differentiation-stage-specific genes [7, 31, 37], and postulate that it plays an important role in gastric cell differentiation through activating of *PGC* and *CCDC28B*.

SET7/9 is known to inhibit cell cycle and apoptosis through methylating of p53 and pRB as non-histones [9, 10, 38]. Taken together with our present finding, loss of SET7/9 expression may promote the tumor progression through absences of H3K4me1 and methylation of non-histones in carcinogenesis, and hence restoration of SET7/9 expression could contribute to new therapeutic strategies for GCs. On the contrary, SET7/9 inhibitors are suggested to be new therapeutic strategies for hormone-dependent breast cancers, such as ERa-positive ones [13, 39], even though there have been no reports on the SET7/9 mutation and expression change in primary breast cancer. Therefore, SET7/9 may play distinct roles in tissue- and cancer-specific manners.

In conclusion, our data provide evidence that SET7/9 has tumor suppressor functions in GCs, and suggest that loss of it may contribute to GC progression and aggressiveness. We also indicated the importance of abnormal gene regulatory mechanisms of loss of SET7/9, that is, a SET7/9-SREK1IP1-MMPs axis, for tumor invasion and migration. Furthermore, it is possible that loss of SET7/9 expression may serve as a potential diagnostic biomarker and a therapeutic target of GCs.

## MATERIALS AND METHODS

### Cell lines and tissue samples

We used 12 GC cell lines, AGS, GCIY, HSC43, HSC44PE, HSC57, HSC60, NUGC4, KATO-III, MKN7, MKN45, MKN74 and TGBC11TKB, which were obtained as described previously [40, 41]. HSC44PE, HSC57 and HSC60 cells were obtained from Dr. Kazuyoshi Yanagihara (National Cancer Center, Japan). All the cell lines were grown in Dulbecco's modified Eagle's medium (DMEM) or RPMI1640 medium containing 10% fetal bovine serum (FBS), and 1% penicillin and streptomycin.

A total of 376 primary GCs from formalin-fixed paraffin-embedded (FFPE) tissue microarray samples in Seoul National University (SNU) were used for immunohistochemistry. In addition, a total of other 25 primary GCs of frozen and matched FFPE tissues were obtained from the affiliated hospital of Tokyo Medical and Dental University (TMDU) in order to compare SET7/9 expression at mRNA and protein levels. Informed consent was obtained from all the patients, and these studies were approved by the Medical Research Ethics Committee for Genetic Research of TMDU and by the Institutional Review Board for Human Subject Research at SNU Hospital.

### RNA extraction, reverse transcription (RT)-PCR and quantitative RT-PCR (qRT-PCR)

Total RNA was extracted using Trizol reagent (Thermo Fisher Scientific Inc., Waltham, MA) or RNeasy (QIAGEN, Valencia, CA), and then reversely transcribed to cDNA using a Superscript kit III. We amplified all genes examined in this study with multiple cycle numbers (28-38 cycles). After PCR amplification, their expression levels were semi-quantitatively determined in 2-3% agarose gels. By using a longer RT-PCR from *SET7/9* exons 1-8, we searched for large deletions including exon skipping in 12 GC cell lines.

qRT-PCR was conducted with a LightCycler system (Roche Diagnostics, Mannheim, Germany) using DNA Master SYBR Green I, according to the manufacturer's instructions. The 2^nd^ Derivative Maximum method was performed for the determination of concentrations using LightCycler software version 3.5. *GAPDH* (*Glyceraldehyde 3-phosphate dehydrogenase*) was used as an internal control for RT-PCR. At 48 hrs after transfection, cells were harvested, and then conventional RT-PCR and qRT-PCR were performed. The representative primer sequences and their PCR conditions are shown in Supplementary Table S1.

### Gene mutation and methylation analyses

The methods of PCR-single strand conformation polymorphism (SSCP) and methylation-specific PCR (MSP) are shown in Supplementary Information. The representative primer sequences and their PCR conditions are shown in Supplementary Tables S1 and S2.

### Transfection

The SET7/9 expression-high GC cell lines, MKN74 (5×10^5^ cells/well), MKN45 (3×10^5^/well), AGS (1×10^5^/well), and KATO-III (2×10^5^/well), were transfected with small interfering RNA (siRNA) of *SET7/9* (SASI_Hs01_00123130 and SASI_Hs01_00123132; Sigma-Aldrich Japan, Ishikari, Japan) or negative control (Mission siRNA Universal Negative Control; Sigma-Aldrich Japan) to give a final concentration of 50nM using a Neon electroporation system (Thermo Fisher Scientific Inc., Waltham, MA). In addition, *SREK1IP1* (SASI_Hs01_00221119 and SASI_Hs01_00221122), *PHF21A* (SASI_Hs02_00322973) and *CCDC28B* (SASI_Hs02_00356628) siRNAs (Sigma-Aldrich Japan) and *PGC* siRNA (SI00683144, QIAGEN) were used.

MKN7 cells were transfected with 2 μg of the wild type-SET7/9-3xFLAG expression vector [9] or the empty vector by electroporation. At 48-72 hrs after transfection, transfected-cells were harvested, and then used for RNA and protein analyses.

### Immunohistochemistry

Mouse monoclonal anti-SET7/9 antibody (clone 5F2.3, 04-0805; Merck Millipore, Darmstadt, Germany) was diluted at 1:100. Immunohistochemistry of SET7/9 protein was performed using a Histofine Simple Stain MAX PO system (Nichirei Biosciences Inc., Tokyo, Japan) and a Ventana BenchMark XT automatic immunostainer system (Ventana Medical Systems, Tucson, AZ) according to the manufacturer's protocol. We assessed the SET7/9 staining score as to both the percentage of the extent of cell staining and intensity. Briefly, immunoreactivity for SET7/9 was scored as follows; proportional score (PS): 0 (negative), 1 (1 - 5% of tumor cells), 2 (6 - 25%), 3 (26-50%), and 4 (51∼100%), and intensity score (IS): 0 (no tumor cell staining), 1 (weak), 2 (moderate), and 3 (strong). The total score (TS) was obtained by summing IS and PS. Because more than half of the tumor cells were stained in SET7/9 expression cases, TS ≥ 6 was considered to represent high expression (retained expression) of SET7/9. Thus, we made two groups, SET7/9 retained (high) and loss/weak (low).

### Western blot

Western blot (WB) was performed as described previously [42]. The primary antibodies used in this study were anti-SET7/9 (rabbit monoclonal, C24B1, 1:1000; Cell Signaling Technology, Danvers, MA; and mouse monoclonal, clone 5F2.3, 1:500; Merck Millipore), anti-SREK1IP1 (rabbit polyclonal, 1:200; GeneTex Inc, Irvine, CA), anti-H3K4me1 (No. 39298, rabbit polyclonal, 1:5000; Active Motif, Carlsbad, CA), anti-FLAG (mouse monoclonal M2, 1:10,000; Sigma-Aldrich Japan), and anti-α-Tubulin (mouse monoclonal, 1:400; Santa Cruz Biotechnology, Santa Cruz, CA) antibodies.α-Tubulin was used as an internal control for WB.

### Microarray analysis

At 48hrs after transfection of *SET7/9* siRNA, total RNA was extracted from MKN74 and MKN45 cells using Trizol reagent. DNA microarray analysis was carried out by DNA Chip Research Inc. (Kanagawa, Japan) using the Agilent Human GE 4x44K v2 Microarray (Design ID: 026652). The raw data have been deposited in NCBI Gene Expression Omnibus (GEO) under the accession number GSE59834.

### Chromatin immunoprecipitation (ChIP)

We prepared sonicated DNA samples from AGS and MKN7 cells using a ChIP-IT Express (No. 39163, Active Motif), and then incubated with 1-3 μg of three histone H3 methylation-related (H3K4me3, No. 39915; H3K9me3, No. 29766; and H3K27me3, No. 39155; Active Motif) and two histone acetylation-related antibodies (acetyl-histone H3 (K9 and K14), #06-599, acetyl-histone H4 (K5, K8, K12, K16), #06-866; Upstate). Moreover, after transfection of the *SET7/9* expression vector or its siRNA into GC cells, ChIP assays were conducted using anti-H3K4me1 polyclonal (ab8895, Abcam, Cambridge, UK) and anti-FLAG monoclonal (Sigma-Aldrich Japan) antibodies. Histone H3 (No. 39163, Active Motif) and Normal Rabbit IgG (No. 2729, Cell Signaling Technology) were used as positive and negative controls, respectively. Input DNA samples were used as internal controls. The primer sequences and their conditions are shown in Supplementary Table S2.

### Cell growth, migration and invasion

We transfected siRNA of *SET7/9* or *SREK1IP1*, or a negative control into MKN74 (3×10^3^ cells) MKN45 (3×10^3^), AGS (0.5×10^3^), and KATO-III (0.5×10^3^) cells on 96-well plates. Cell proliferation was evaluated on days 1, 3, 5 and 7 after replating by determining the number of cells with a Cell Counting Kit-8 (Dojindo, Kumamoto, Japan), according to the manufacturer's instructions [42].

The siRNA-transfected GC cells (1×10^4^ cells) were grown in 24-well transwells (8 μm pore size)-coated with (invasion) or without (migration) matrigel (BD Biosciences, Franklin Lakes, NJ) [43]. After 18-48 hrs, invading cells were detected by Diff-Quik staining. The method of scratch assay is shown in Supplementary Information.

### Statistics

All the experiments were repeated at least twice with duplicate or triplicate samples. The Mann-Whitney's U-test, t-test and χ^2^-test were used to compare the values for the test and control samples. The nonparametric Mann—Whitney U-test was used to determine the differences between the cases with SET7/9-expression high and low groups. A value of *P* < 0.05 was taken as being significant. Patient survival was calculated from the date of surgery until death or the date of last follow-up. Survival was evaluated by the Kaplan-Meier method, and uni- and multi-variate survival analyses were performed using the Cox proportional hazard model.

## SUPPLEMENTARY FIGURES AND TABLES







## Abbreviations

CGI: CpG island
ChIP: Chromatin immunoprecipitation
DFS: disease-free survival
GC: gastric cancer
HMT: histone methyltransferase
H3K4me1: histone H3 lysine 4 mono-methylation
IHC: immunohistochemistry
MMP: matrix metalloproteinase
MSP: methylation-specific polymerase chain reaction
OS: overall survival
(q)RT-PCR: (quantitative) reverse transcription-polymerase chain reaction
TSGs: tumor suppressor genes
TSS: transcriptional start site.
