# Supplementary material for: Reduced expression of SET7/9, a histone mono-methyltransferase, is associated with gastric cancer progression
Source: Oncotarget. 2015 Dec 19;7(4):3966–83. doi: 10.18632/oncotarget.6681 (PMC4826183; doi:10.18632/oncotarget.6681)
Supplement: Supplementary file 1 [file oncotarget-07-3966-s001.pdf]

SUPPLEMENTARY FIGURES AND TABLES

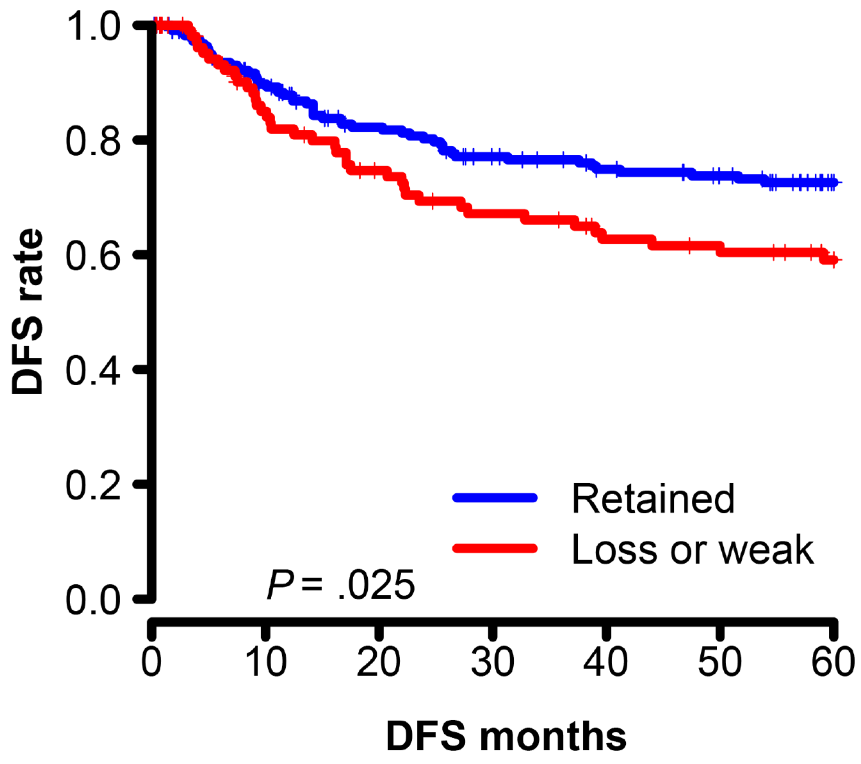

Supplementary Figure S1: Kaplan Meier survival curve of disease free survival of GC patients with reference to SET7/9 expression. The disease-free survival rate of the GC patients showing loss/weak SE7/9 expression was significantly lower than those of the GC patients with SET7/9 expression-positive GCs ($P = 0.025$, logrank test).

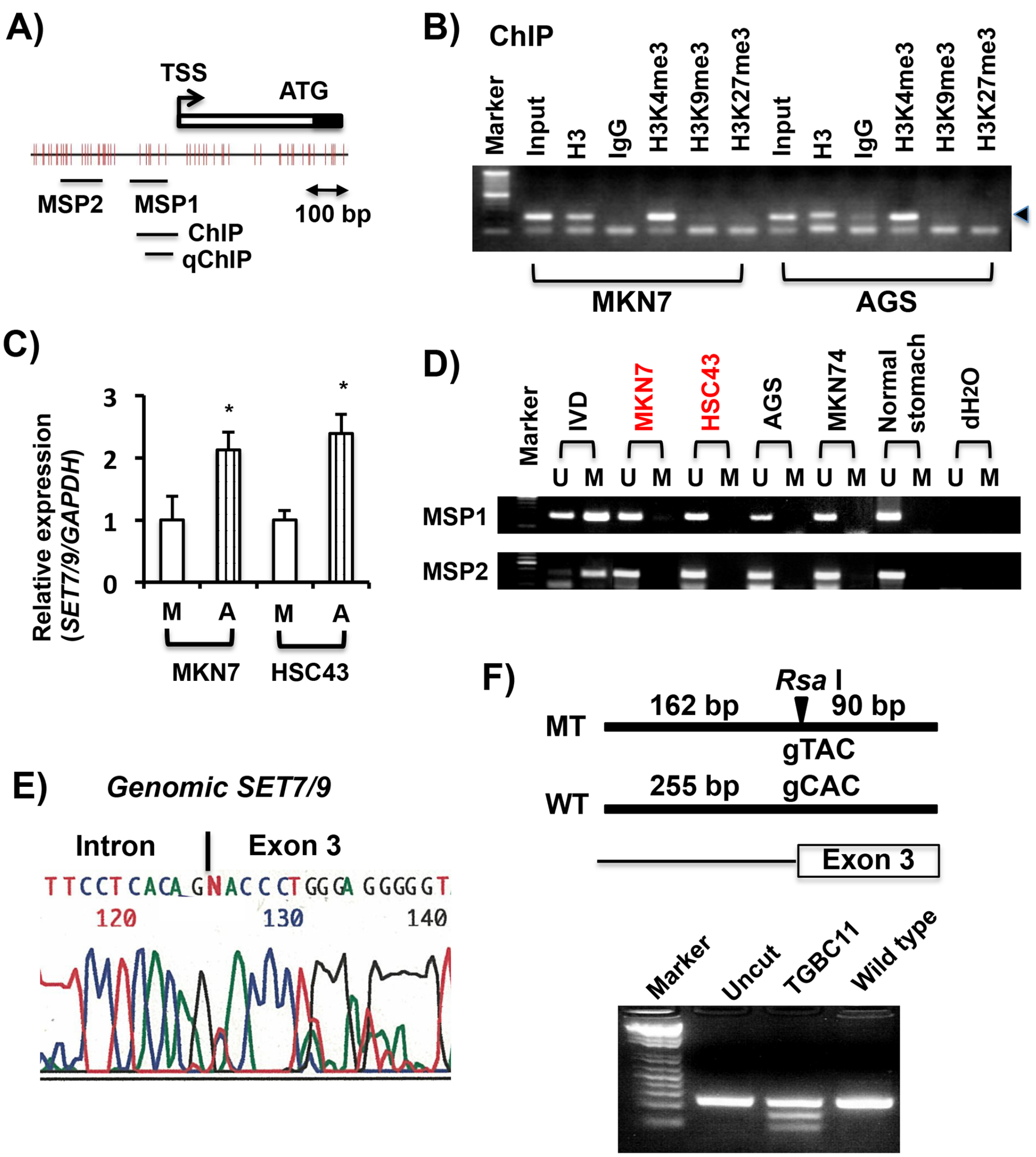

Supplementary Figure S2: Analyses of *SET7/9* alterations in GC cells. A. Schematic representation of the 5′-region of *SET7/9* (Genbank accession No. NW_004078021). A box indicates exon 1, including coding (black) and non-coding (white) regions. Vertical bars show CpG sites. Bars below the CpG sites indicate the regions subjected to methylation-specific PCR (MSP) and chromatin immunoprecipitation (ChIP). **B.** ChIP assay of the *SET7/9* promoter region in AGS and MKN7 cells exhibiting strong and weak *SET7/9* expression, respectively. ChIP assay was performed with anti-H3K4me3, H3K9me3 and H3K27me3 polyclonal antibodies. Histone H3 and IgG were used as positive and negative controls, respectively. The PCR products were loaded onto 2.5% agarose gels. Input DNA amplified from sonicated DNA samples were used as an internal control. **C.** qRT-PCR analysis of *SET7/9* expression in GC cells. MKN7 and HSC43 cells were daily treated with 5 (M 5-aza-2′ deoxycytidine (5-aza-dC; Sigma-Aldrich, St Louis, MO) for 72 hrs. M, mock; A, 5-aza-dC. $*P < 0.01$. **D.** MSP analyses of the CpG islands in the 5′ region of *SET7/9* in 4 GC cell lines and a non-cancerous stomach tissue. Although MKN7 and HSC43 cells exhibited up-regulation of *SET7/9* expression by 5-aza-dC treatment, CGI methylation was not found at two MSP sites (MSP1 and MSP2). *In vitro*-methylated DNA (IVD) was used as a positive control of MSP. The PCR products recognizing unmethylated (U) and methylated (M) CpG sites were loaded onto 2.5% agarose gels. The primer sequences and the PCR conditions for RT-PCR, MSP and ChIP are shown in Supplementary Tables 1 and 2. **E.** Sequencing analysis of genomic *SET7/9* exon 3 in TGBC11TKB cells. **F.** A heterozygous C to T transition at the first nucleotide of *SET7/9* exon 3 in TGBC11TKB cells created a new *Rsa* I (GTAC) digestion site. The PCR product from TGBC11TKB cells showed *Rsa* I-digested bands, while the other product without the transition change (WT) did not. An undigested PCR product (Uncut) was used as a control of *Rsa* I digestion. The PCR products were loaded onto a 3% agarose gel. This nucleotide change was detected in only TGBC11TKB DNA but not in other 42 cases examined (2.3%).

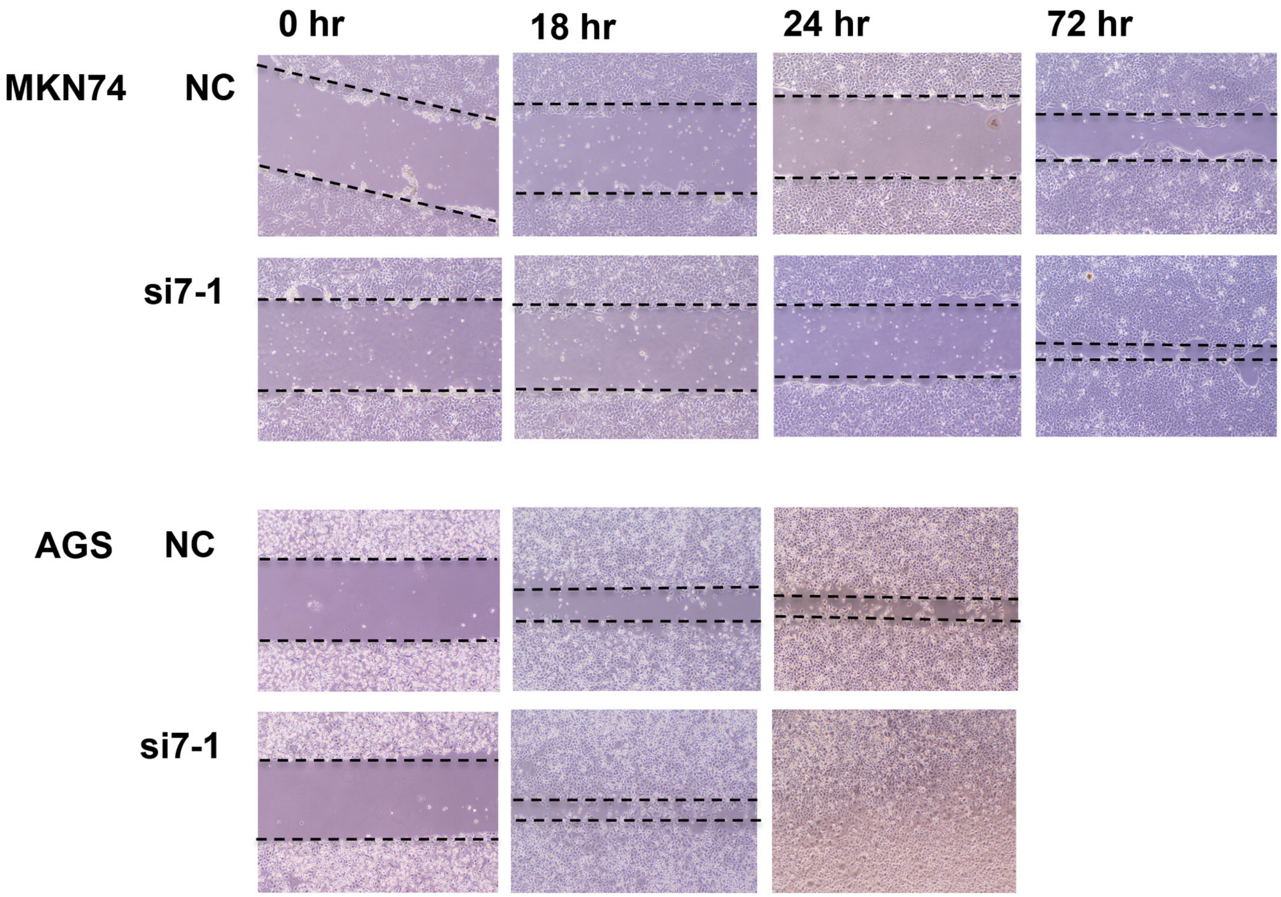

Supplementary Figure S3: Effects of siRNA-based *SET7/9* knockdown in GC cells. Scratch assay of GC cells after SET7/9 knockdown. MKN74 and AGS (5x10^5 cells) with *SET7/9* or negative control siRNA transfection were plated in 6cm dishes and grown until confluent. Scratch assay was performed using a plastic pipette tip. Representative photographs showing the cell migration at 0, 18, 24 and 72 hrs are shown.

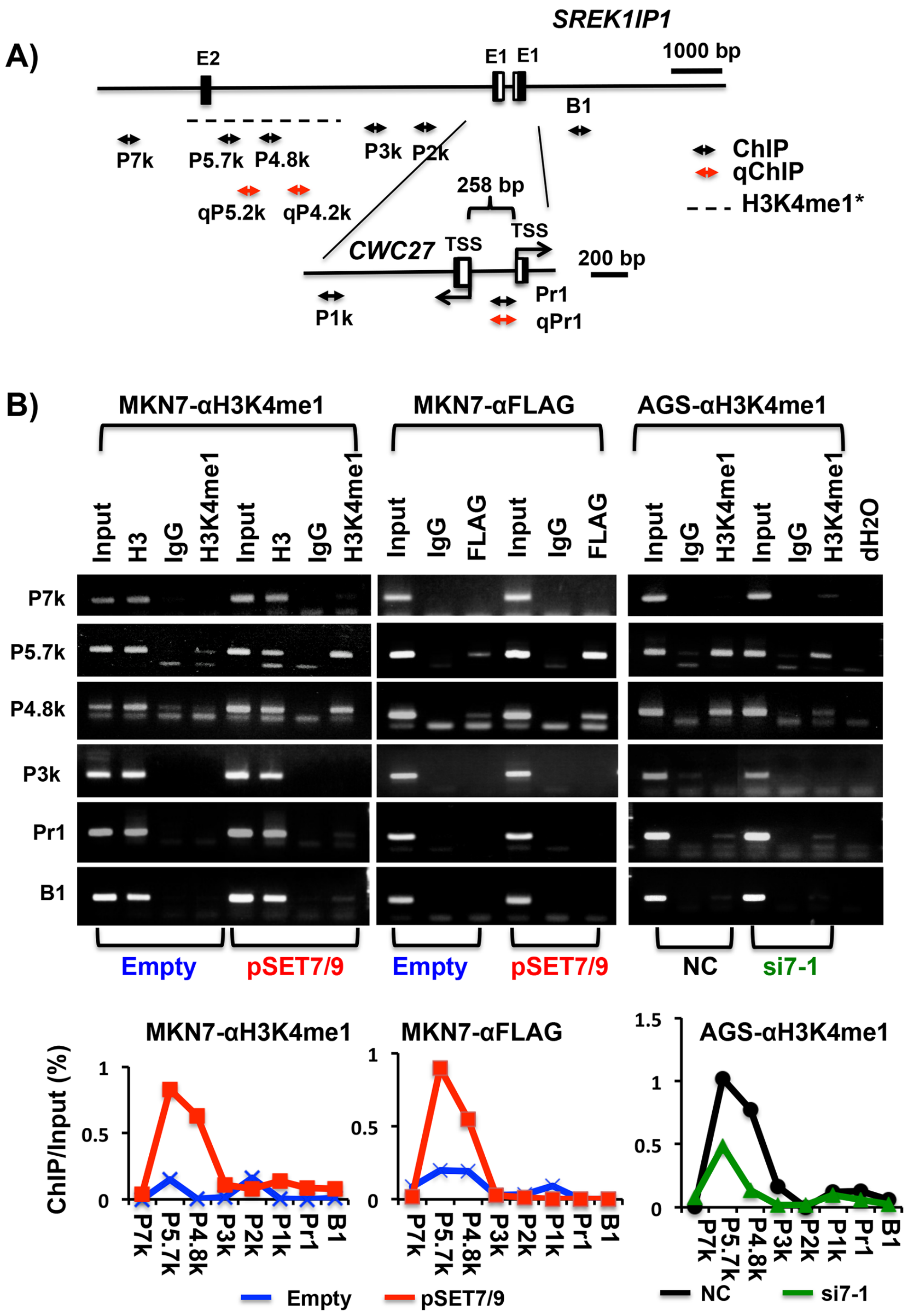

Supplementary Figure S4: The relationship between SET7/9 and H3K4me1 at the 5′-upstream region of *SREK1IP1* in GC cells. A. Schematic representation of the 5′ region of *SREK1IP1*. The database of the UCSC Genome Bioinformatics Site indicates that an approximately 4–6kb upstream region from *SREK1IP1* is enriched with H3K4me1 (dotted line), which is known to be a histone mark for active genes (3,32). Seven regions of 5′-upstream of the *SREK1IP1* transcriptional start site (TSS) and one 3′-region were amplified using primer sets (Supplementary Table 2). Closed boxes indicate exons and horizontal arrows indicate ChIP sites amplified in this study. **B.** Top: Representative data obtained on ChIP assays of *SREK1IP1*. After overexpression of SET7/9 in MKN7 cells, ChIP assay was performed using anti-H3K4me1 (left panel) and anti-FLAG (middle) antibodies. The H3K4me1 level was determined in AGS cells with *SET7/9* knockdown (right panel). Anti-Histone H3 and normal rabbit IgG were used as positive and negative controls, respectively. The PCR products were loaded onto 2.5% agarose gels. Input DNA amplified from each sonicated DNA sample was used as an internal control. Bottom: The intensity of the PCR products of ChIP assays was semi-quantitatively determined using Image J 1.47v software. (***Continued***)

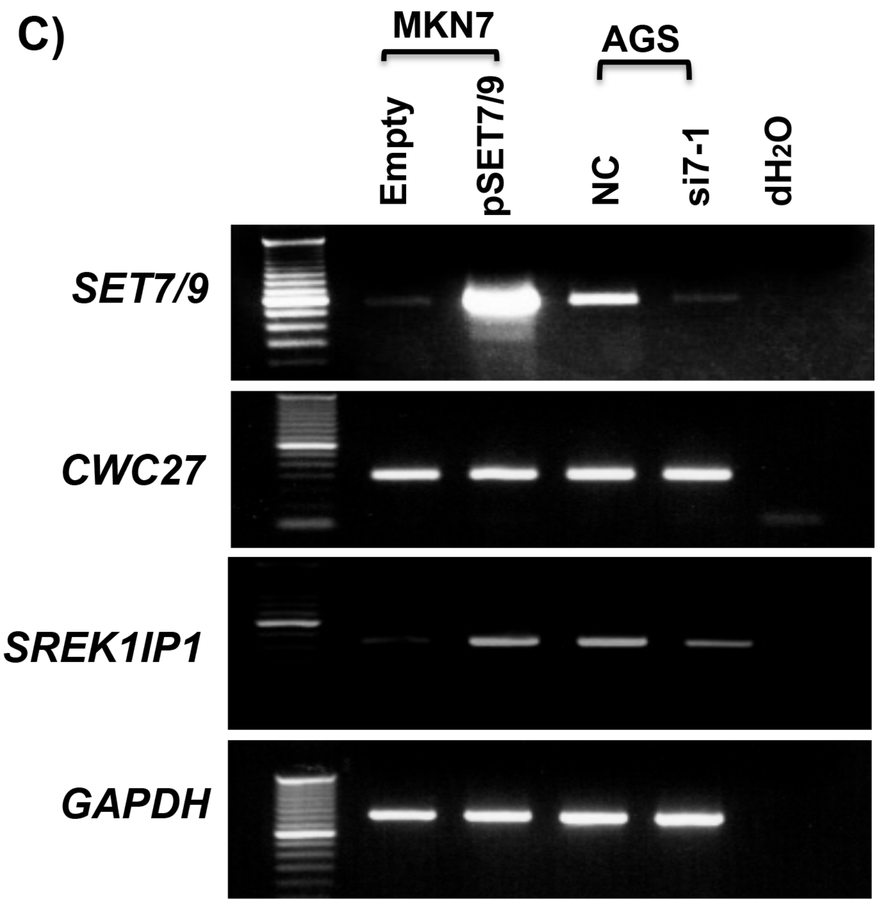

Supplementary Figure S4: (*Continued*) The relationship between SET7/9 and H3K4me1 at the 5′-upstream region of *SREK1IP1* in GC cells. C. RT-PCR analyses were performed in order to determine whether or not *SET7/9* can regulate *CWC27* expression as well as *SREK1IP1*. Neither overexpression nor knockdown of *SET7/9* caused expressional changes of *CWC27* in GC cells. The PCR products were loaded onto 2% agarose gels. *GAPDH* was used as an internal control.

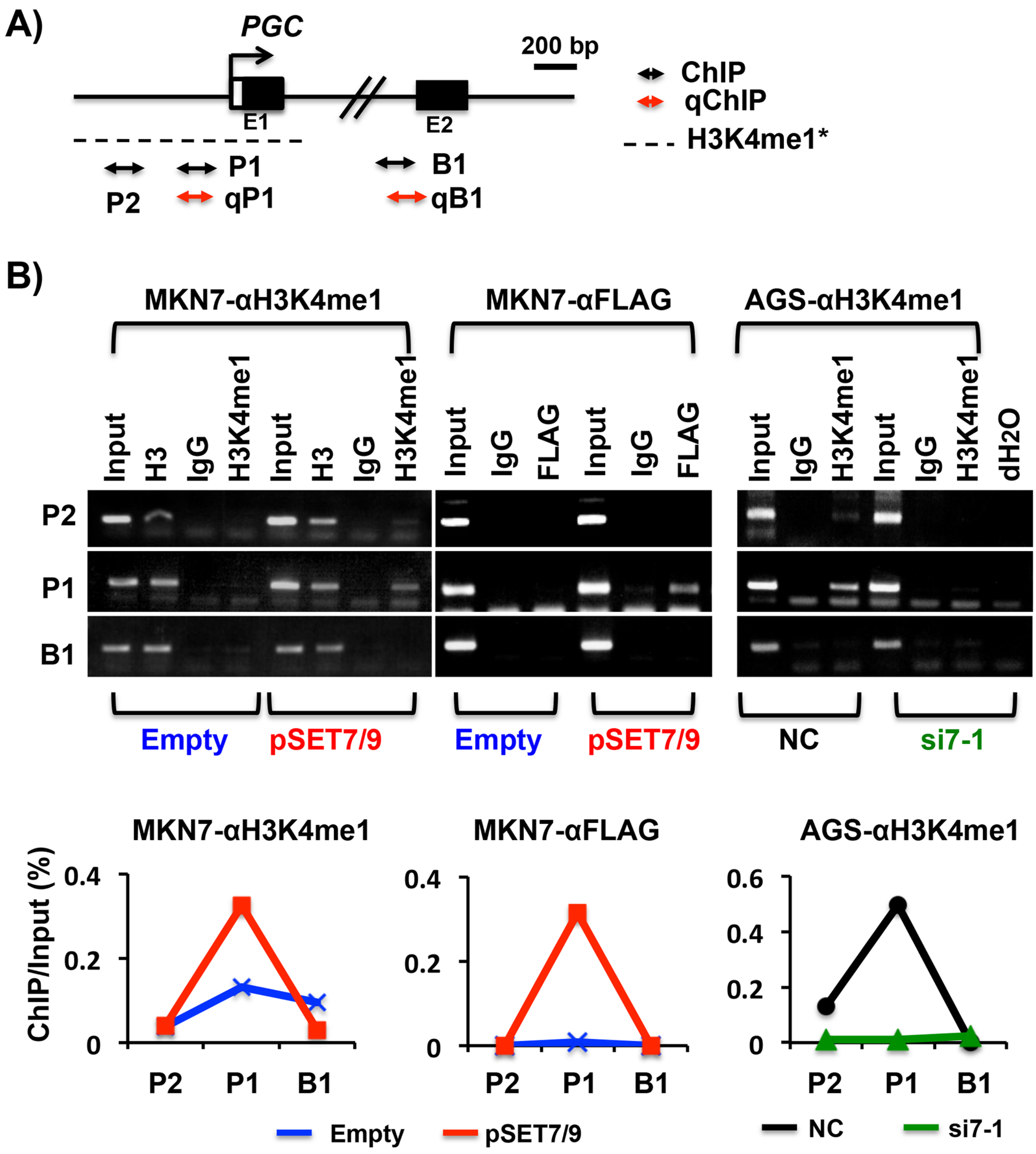

Supplementary Figure S5: The relationship between SET7/9 and H3K4me1 at the PGC promoter in GC cells. A. Schematic representation of the *PGC* promoter region. Horizontal arrows indicate ChIP sites amplified in this study. Three regions (P1, P2 and B1) were amplified using primer sets (Supplementary Table 2). **B.** ChIP assays (top) and its semi-quantitative analyses (bottom) were conducted using anti-H3K4me1 and anti-FLAG antibodies for GC cells with SET7/9 overexpression or knockdown, in which methods are similar to those of Supplementary Figure 4.

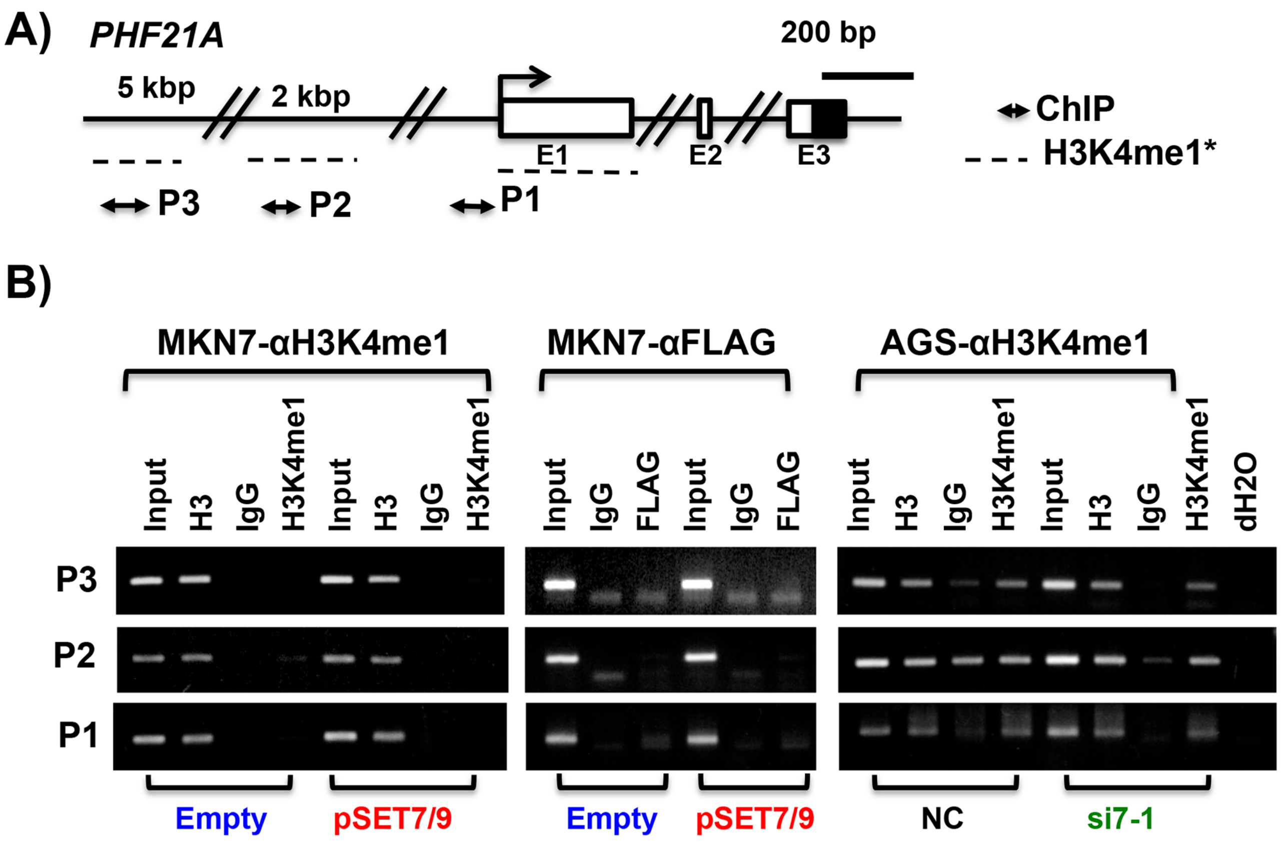

Supplementary Figure S6: Analysis of H3K4me1 at the 5′-upstream region of *PHF21A* in GC cells. A. Schematic representation of the 5′-upstream region of *PHF21A*. Since ChIP-seq data on the UCSC database exhibited H3K4me1 enrichment at the three sites (dotted line), we examined these three regions (P1, P2 and P3) in GC cells by ChIP assays. **B.** ChIP assays was conducted using anti-H3K4me1 and anti-FLAG antibodies for GC cells with SET7/9 overexpression or knockdown, in which methods are similar to those of Supplementary Figure 4.

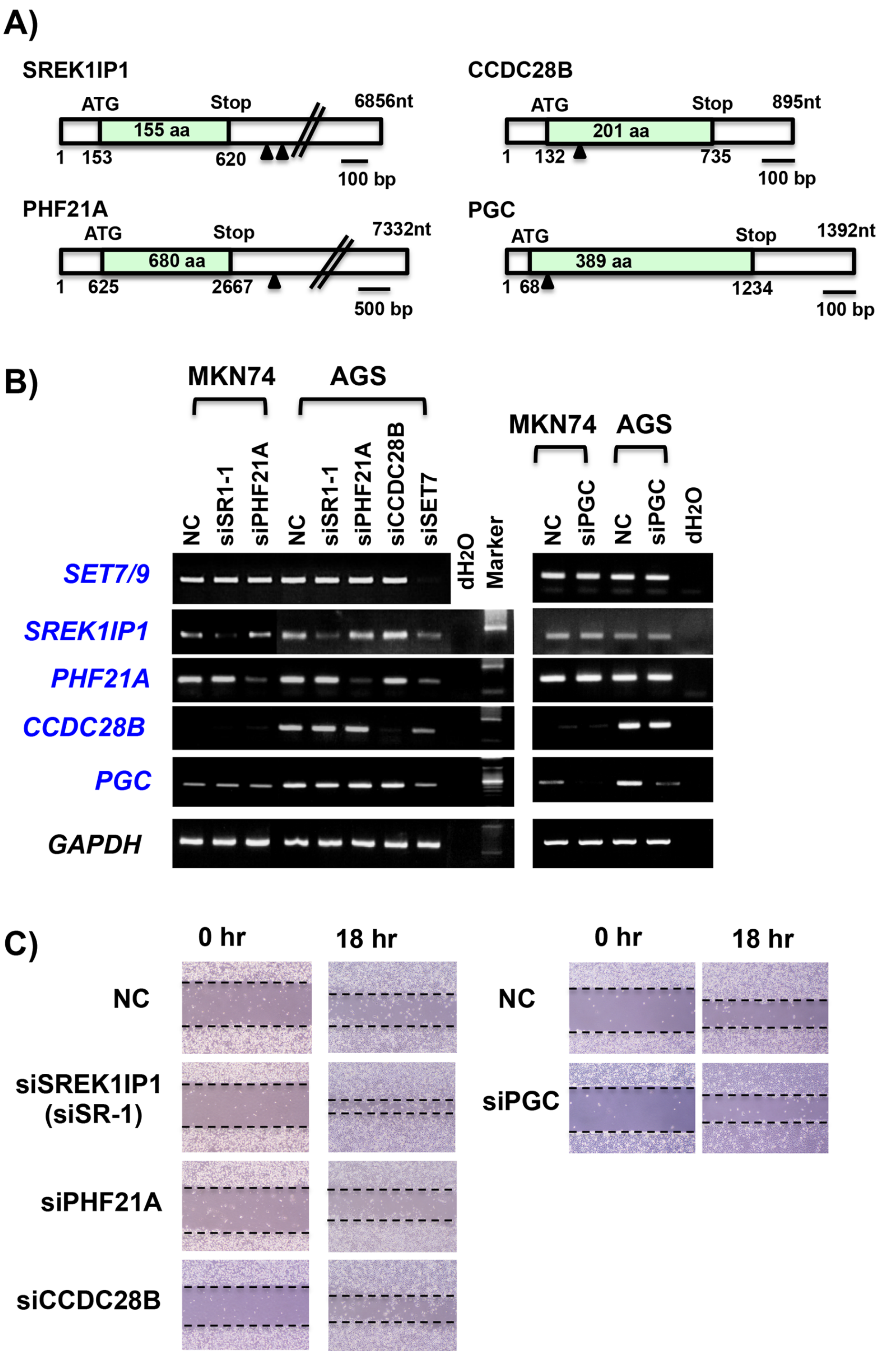

Supplementary Figure S7: Knockdown of the *SET7/9* target genes in GC cells. A. Locations of *SREK1IP1*, *PHF21A*, *CCDC28B* and *PGC* siRNAs used in this study. **B.** RT-PCR analyses of the *STE7/9* target genes in GC cells with their siRNAs transfection. At 48 hrs after transfection of *SREK1IP1, PHF21A*, *CCDC28B* or *PGC* siRNA to MKN74 and AGS cells, the effects of the siRNA transfection were analyzed by RT-PCR. The PCR products were loaded onto 2% agarose gels. *GAPDH* was used as an internal control. Each siRNA specifically inhibited its target gene, but there was no effect on *SET7/9* expression in these two GC cell lines, indicating that there are no feedback effects on *SET7/9*. **C.** Scratch assays of AGS cells with transfection of siRNA of the *SET7/9* target genes, *SREK1IP1, PHF21A*, *CCDC28B* and *PGC*. Cell migration was faster in AGS cells with *SREK1IP1* siRNA transfection compared to ones with the negative control and other two siRNAs. Representative photographs showing the cell migration at 0 and 18 hrs are shown.

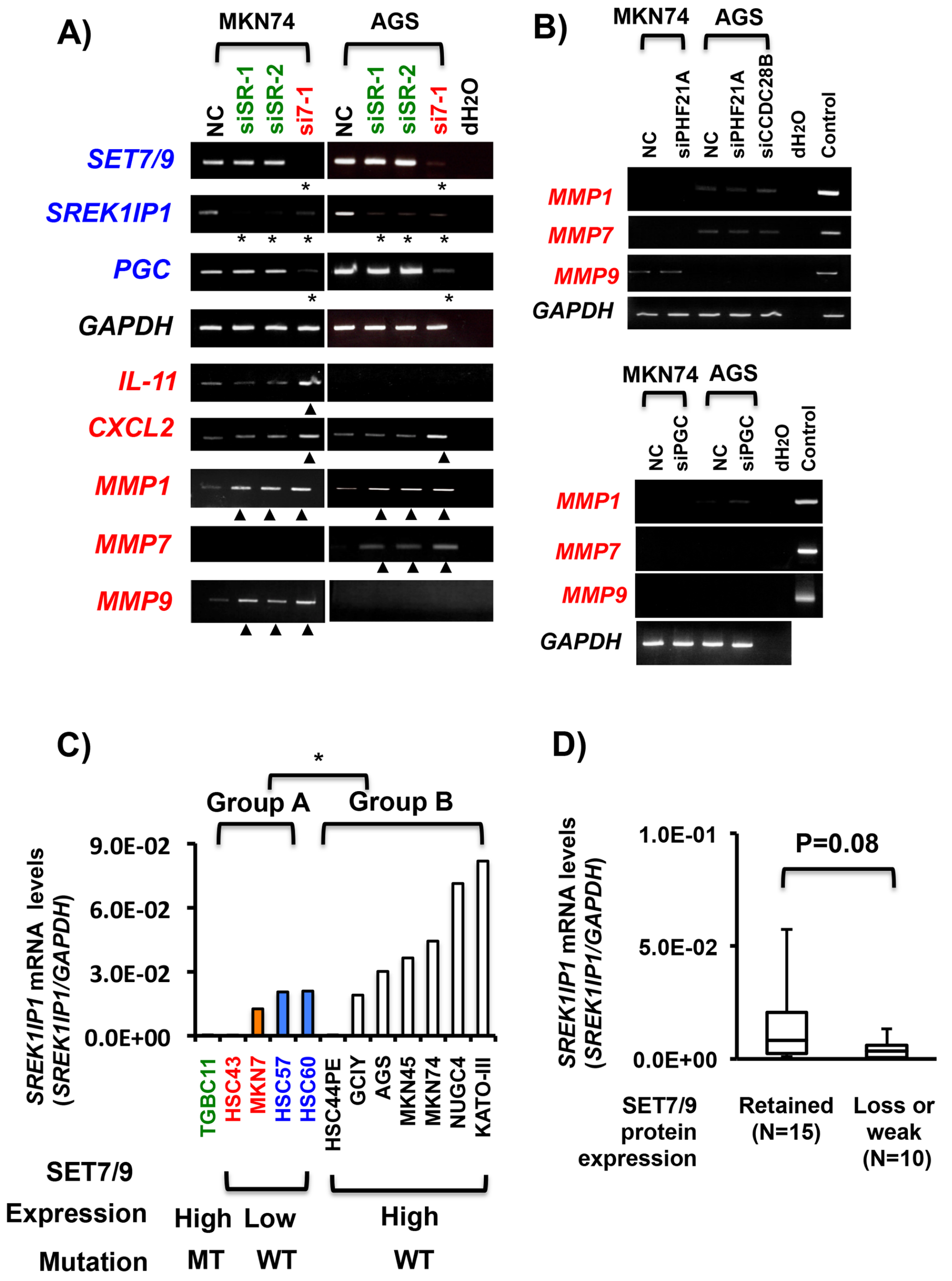

Supplementary Figure S8: The effects of *SREK1IP1* knockdown in GC cells. A. RT-PCR analyses of the *SET7/9* target genes in MKN74 and AGS cells with *SREK1IP1* siRNA transfection. *SET7/9* siRNA was used as a positive control in this experiment. PCR products showing down- and up-regulation of the *SREK1IP1* target genes are indicated by asterisks and triangles, respectively. **B.** Knockdown of *PHF21A*, *CCDC28B* and *PGC* did not show any effects on expression of three MMP genes in GC cells. **C.** qRT-PCR analysis of *SREK1IP1* in 12 GC cell lines. These GCs were divided into two groups according to the SET7/9 alteration status. Group A is SET7/9 alteration-positive (SET7/9-mutated TGBC11TKB cells and four cell lines with low SET7/9 expression) and group B is its-negative (remaining seven cases with high SET7/9 expression and no mutation) (Figure 2B). The average (column) of two independent experiments on GC cell lines is indicated. Relative expression was calculated using *GAPDH* expression as an internal control. The expression level of *SREK1IP1* in Group A was significantly lower than that in Group B (Mann–Whitney *U*-test, *$P = 0.033$). **D.** *SREK1IP1* expression in primary GC tissues. The *SREK1IP1* expression levels were compared between the GC tissues that were SET7/9 protein expression-positive (retained, $N = 15$) and -negative (loss or weak, $N = 10$). Relative expression was calculated using *GAPDH* expression as an internal control, and the average (column) ± S.D (bar) is indicated. Mann–Whitney *U*-test, *$P = 0.073$.

Supplementary Table S1: Representative primer sequences and their PCR conditons in this study.

Supplementary Table S2: Primer sequences and the ChIP-PCR conditons used in this study.

SUPPLEMENTARY MATERIALS AND METHODS

PCR-SSCP

Genome DNA was extracted by the standard phenol–chloroform procedure, and PCR-SSCP was performed as described previously [45]. We screened genetic alterations of *SET7/9* in 12 GC cell lines and 25 primary GCs. If abnormal SSCP patterns were observed, the PCR products were purified with a QIA-quick spin PCR purification kit and then subjected to a TA-cloning and/or direct sequencing. The primer sequences and the PCR conditions are available from the authors on request.

Methylation analysis

Bisulfite treatment of genomic DNA and the following methylation-specific PCR (MSP) were performed as described previously [46]. We examined the methylation status at the *SET7/9* promoter region with a dense CpG island in 12 GC cell lines and 25 primary GCs. The primer sequences and the PCR condition analyses are shown in Supplementary Table 2.

Scratch assaying

After transfection of siRNA of *SET7/9, PHF21A* or *CCDC28B* in AGS (5x10^5/6cm-dish), cells were grown in RPMI1640 containing 10% FBS until confluent (24 hrs). MKN74 (5x10^5/6cm-dish) cells were transfected with *SET7/9* siRNA and grown until confluent (>74 hrs). Negative control siRNA was also transfected to these cells. A monolayer of confluent cells was then scratched manually using a plastic pipette tip, and then grown for 18–24 hrs [47].

REFERENCES

1. Akiyama Y, Yagi OK, Ishikawa T, Nagasaki H, Saitoh K, Yuasa Y. Genetic alterations are frequent in APC but rare in the TGF-beta type II receptor gene in cancer in adenomas of the colon. Cancer Lett 1998;125:89–96.
2. Herman JG, Graff JR, Myöhänen S, Nelkin BD, Baylin SB. Methylation-specific PCR: a novel PCR assay for methylation status of CpG islands. Proc Natl Acad Sci USA 1996;93:9821–9826.
3. Liang CC, Park AY, Guan JL. In vitro scratch assay: a convenient and inexpensive method for analysis of cell migration in vitro. Nat Protoc 2007;2:329–333.
